# Lignin, the Lignification Process, and Advanced, Lignin-Based Materials

**DOI:** 10.3390/ijms241411668

**Published:** 2023-07-19

**Authors:** Maria Balk, Pietro Sofia, Axel T. Neffe, Nicola Tirelli

**Affiliations:** 1 Institute of Functional Materials for Sustainability, Helmholtz-Zentrum Hereon, Kantstrasse 55, 14513 Teltow, Germany; maria.balk@hereon.de; 2 Laboratory of Polymers and Biomaterials, Fondazione Istituto Italiano di Tecnologia, Via Morego 30, 16163 Genova, Italy; pietro.sofia@iit.it; 3 The Open University Affiliated Research Centre at the Istituto Italiano di Tecnologia (ARC@IIT), Via Morego 30, 16163 Genova, Italy

**Keywords:** lignin, lignols, lignification biochemistry, wood ultrastructure, healthcare application, supercapacitors, shape-memory materials

## Abstract

At a time when environmental considerations are increasingly pushing for the application of circular economy concepts in materials science, lignin stands out as an under-used but promising and environmentally benign building block. This review focuses (A) on understanding what we mean with lignin, i.e., where it can be found and how it is produced in plants, devoting particular attention to the identity of lignols (including ferulates that are instrumental for integrating lignin with cell wall polysaccharides) and to the details of their coupling reactions and (B) on providing an overview how lignin can actually be employed as a component of materials in healthcare and energy applications, finally paying specific attention to the use of lignin in the development of organic shape-memory materials.

## 1. Introduction

Lignin is a naturally abundant polymeric material. As a major component of the walls of plant cells (see Section 2), it is virtually ubiquitous and is estimated to be produced by plants at the tune of around 20 billion tons/year [1]; this makes lignin one of the most common macromolecular systems on our planet. Although naturally it is always intermixed with other biomass components, a number of processes—such as the extraction of polysaccharides (chiefly cellulose) to yield paper or textile fibers or the production of bioethanol—provide lignin as a byproduct, making it in principle usable for other downstream applications. For example, in the mid-2010s paper/pulp production alone produced 50–70 million tons of lignin p.a. [2]. The growing use of biofuels suggests that bioethanol production can be an even larger lignin source in a very close future.

Chemically, lignin is a (variably) cross-linked polyphenol; it is produced during the radical/oxidative oligo- or polymerization of a variety of compounds, which predominantly share a common *p*-hydroxycinnamic skeleton. Section 3 specifically focuses on the identity of lignin precursors and on the mechanisms presiding their polymerization, which needs to be understood in detail to rationalize structure and properties of lignin-based materials (including wood) and to potentially engineer them by design.

Despite our reasonably good grasp over the synthesis, structure, and natural role of lignin and notwithstanding its very wide availability, its exploitation has always been an issue. Firstly, its chemical identity (molar mass distribution, monomer composition, and degree of branching) is quite variable and sometimes difficult to control. Secondly, lignin has always been considered a low-value waste material: its thermo-mechanical properties are unattractive, making its processing difficult, and despite being a natural material, it is poorly degradable and can also reduce the degradability of materials it is connected to, such as polysaccharides. Indeed, lignin typically reduces their digestibility in animals and more in general hampers the direct transformation of plant biomasses into biofuels or sugars through (bio)chemical methods [3] or pyrolysis [4]. Currently, only about 2% of the produced lignin finds commercial use, predominantly in specialty chemicals [2], while the rest is being either burned or added to animal fodder essentially as a bulking agent.

Notwithstanding these issues, its inherently sustainable origin and low production costs (from tens to hundreds USD/ton, depending on the purity [1]) are both undeniable advantages and powerful drives for the development of new materials based on lignin. An additional advantageous factor is that the costs associated with the disposal of a byproduct disappear when lignin is used as a building block for added-value materials, which lends even more economic sense to this perspective.

The following lines list the three main approaches to lignin processing, on the one hand highlighting those discussed in the present review and on the other hand pointing the reader to literature reviews for all others:

(A) Engineering the lignification process in order to modify lignin composition, in this way to improve its fermentability to produce biofuels [5] or its digestibility in animal fodder [6] or to allow its easier incorporation into biomaterials [7]. Although this is not the focus of this review, these points are touched upon at the end of Section 3.4.

(B) Production of high(er)-value chemicals (e.g., vanillin [8], flavonoids [9], and many other (methoxy)phenols and catechols and non-aromatic carboxylates and ketones) via thermal, reductive, oxidative, or basic/acid-catalyzed depolymerization. Here, this approach is not discussed in detail, and we refer the reader to excellent and specialized recent reviews [10,11].

(C) Use of lignin (directly in its native form or after chemical derivatization at phenol/alcohol OH groups) in a variety of materials, where lignin (1) undergoes dramatic chemical changes, e.g., being converted into carbonaceous materials via graphitization [12]; (2) becomes a building block for macromolecular products such as polyurethanes [11], which then undergo their own processing; and (3) is integrated in nanostructured materials as a functional component. Section 4 reviews the third case, specifically focusing on application areas that confer lignin the highest added value, i.e., healthcare (Section 4.1), energy storage (Section 4.2), and shape-memory materials (Section 4.3).

## 2. Plants, Wood, and Where to Find Lignin

Wood is the prototypical lignified tissue. The following discussion focuses therefore on wood, but the reader should bear in mind that not all plants contain wood; for example, grasses are non-woody plants. However, virtually all plants (bar bryophytes) produce lignin and have lignified tissues that bear significant structural and biochemical similarities to wood, although with important compositional and mechanical differences.

The defining feature of wood and lignified tissues is to be found at a (supra)molecular level: their hardness is mostly related to the presence and the structural details of interconnected three-dimensional networks, which are made of two classes of partially phase-separated macromolecular components: polysaccharides such as cellulose, hemicelluloses, and pectins and the rather ill-defined, part-aromatic material referred to as lignin. Polysaccharides usually make up 65–75% of wood’s weight; lignin makes up 18–35% [13]. The relative ratio of these components is used to classify trees into the limit definitions of softwood (typically from gymnosperms, i.e., non-flowering trees) [14] and hardwood (typically from angiosperms, i.e., flowering trees [15]). Coniferous species are typical representatives of gymnosperms, and their softwood is made up of 40–45 wt% cellulose, 26–34 wt% lignin, and 7–14 wt% of other (cell wall) polysaccharides; the hardwood of deciduous species has a similar cellulose content (38–49 wt%) but is typically lower in lignin (23–30 wt%) and higher in cell wall polysaccharides (19–26 wt%) [16]. However, many plants are commonly categorized as intermediate types (mixedwoods).

Wood also includes a fraction of low molecular weight (MW) organic compounds (polyphenols such as stilbenoids, flavonoids or tannins, or terpenes [17]) as minor components; their amount typically does not exceed 5–10% of the dry wood mass, but occasionally, most commonly in tropical and sub-tropical plants, they may reach up to 20 wt% [18]. Apart from being the main determinants of wood smell and color, the polyphenolic nature of these molecules makes them capable of scavenging free radicals and also polymerize oxidatively; on the one hand, this confers them a protective role but on the other hand may allow a certain degree of integration in lignification processes, therefore blurring the distinction between these low MW extractives and *strictu sensu* lignin precursors (see Section 3), above all those with flavonoid or stilbenoid structure. All the above, however, should be considered indications rather than precise data: the actual composition of wood depends on a variety of factors, such as the specific location in the plant’s body, its age, and on the environmental conditions the plant has experienced during the production of wood (affecting its biosynthesis) and at later stages (modifying it after its production). For example, polysaccharides may undergo hydrolysis in the long term [19]. It is also noteworthy that herbaceous (=non-woody) plants have lignin too, but in lower amounts than woody plants; their lignin content ranges from 0.4 (maize flour) through 1.2 (white lupins), 3.5 (sugar beet fibers), to up to 12–15 wt% (whole grasses or cereal husks) [20]. The following sections refer mostly to woody plants (because of their higher lignin content), but most information on cell walls and lignification (bio)chemistry applies to both them and non-woody (herbaceous) organisms, and references will be made accordingly.

### Structural Features of Lignified Tissues (Wood)

**Figure 1:**
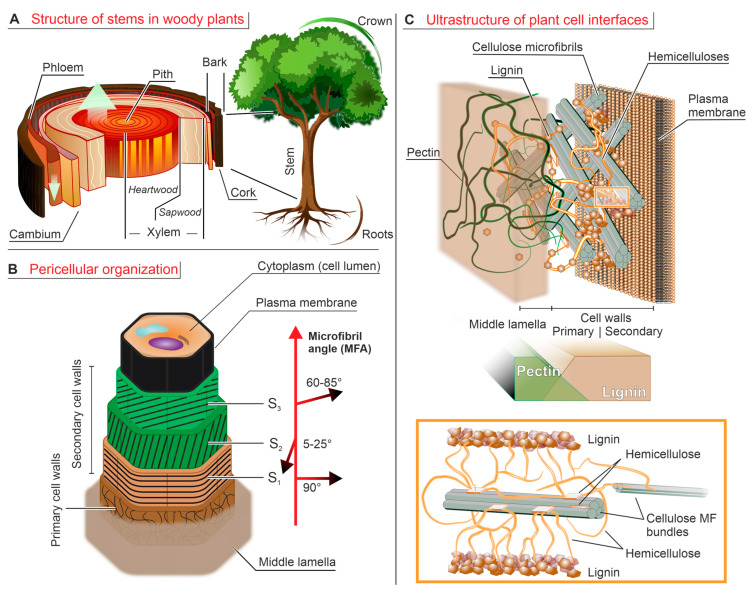
(**A**). Cross-section of a wood trunk. The innermost layer is the spongy pith, surrounded by the inner vascular tissue (xylem); the latter is divided into the inactive heartwood (for mechanical support) and the active sapwood (for long-range water transport). More externally, *cambium* produces both new sapwood and the more external vascular tissue of phloem, and finally the highly lignified bark provides a protective barrier. (**B**). A multi-layer structure wraps around the plant cell cytoplasm (the lumen), starting with the plasma membrane, developing with three layers (S_1_-S_2_-S_3_) of lignin-rich secondary cell walls and ending with the more external primary walls that are surrounded by the pectin-rich middle *lamella*, which also provides connection to adjacent cells. The microfibril angle (MFA) of cellulose varies radially and correlates with the mechanical properties of the layers. (**C**). In cell walls, cellulose microfibrils (MF, gray rods) are embedded in a matrix composed of hemicelluloses (in orange) and pectin (in green, only present in the primary walls). The inset shows how the crystalline domains of hemicellulose bind to cellulose via interfacial hydrogen bonding, while its amorphous chain portions bridge among them and at the same time covalently connect to lignin microparticles (lignin–carbohydrate complexes, LCC). In the better known “tethered network” model, hemicellulose chains fulfill the role of bridges between distant elements (cellulose bundles and/or lignin), but actually crystalline hemicellulose can also bridge cellulose microfibrils from within their bundles, which makes them both mechanically active and inaccessible to hemicellulases [29]. Pectin is the main component of the middle *lamella* and is also abundantly present in the primary cell walls (30–50%) [30] but is basically absent in the secondary ones; i.e., its concentration profile is almost opposite to that of lignin. Please note that the relative thickness of the various layers is not in scale (secondary walls being much thicker than all other elements).

At a macroscopic level, woody plants are structured into roots, stems, and crowns. Here, we will focus on stems (their trunks, Figure 1A), which are the most wood-rich regions in woody plants, but they are also the largest parts of the bodies of herbaceous plants.

Transversally, the stem’s innermost part is referred to as the pith or *medulla*, a spongy and rather soft tissue. Although accurate measurements of its modulus are hard to find, it increases stiffness for very soft stems such as maize stalks (where the pith has the largest volume fraction) [21], while in hard stems such as those of pine trees it has a (minor) softening effect [22]. It is a moderately lignified tissue (8–15 wt% in herbaceous plants such as maize [23] or thistle [24], although occasionally, e.g., in coconut husk, may reach 25 wt% [25]).

Surrounding the pith, stems feature a system of concentric and tapering layers (the annual rings) named xylem, which are enclosed in the external protective multilayer of the wood bark (that contains the phloem, see later). The xylem takes care of the long-range (upward) water-transport from roots to appendages (leaves). Within any annual ring of the xylem, a lighter-colored part named earlywood is distinguished from a darker and denser part delimiting the ring, called latewood; the vascular system of the latter presents smaller cavities, and its cells have thicker, more lignified walls, making it denser and harder than earlywood [26,27]. The xylem rings can be grouped into two distinct concentric regions, sapwood and heartwood. Sapwood (or young xylem) is the younger, softer, physiologically active outer portion of the wood trunk. Besides being the main contributor to the stem mechanical support, sapwood also acts as a storage reservoir for water, as well as for polysaccharides such as starch that reduce water loss [28].

With age, sapwood cells gradually die, and the tissues become darker, producing the central cylinder known as heartwood, distinguished from a rather thin (1 cm at most) transition zone. This cell death causes the local release of many low MW compounds (extractables, also known as extractives, typically polyphenolics), whose oxidative polymerization and integration with lignin darken and provide durability to this xylem area [31,32]. The stem’s growth is regulated by a thin layer located between the xylem and phloem, the vascular *cambium*, which forms a circular front of precursor cells differentiating into both the internal xylem and the external phloem, the latter being responsible for the downward transport of materials from sites of photosynthesis. Taxonomically, the phloem is part of the external layer of the stem, i.e., the bark, which further comprises the outermost cork and an intermediate layer of cork *cambium* (containing cork immature cells). The cork acts as a protective layer against drying and other environmental conditions, as well as pathogens, and is highly lignified and rich in extractives. Detailed comparisons between xylem, phloem, and cork are rather rare, but it can be said that lignification increases in that order, with significant variations also in lignin composition [33,34] (see Section 3.4).

At a cellular level, a structural feature is common to both woody and herbaceous plants: their cells have walls with a helically reinforced, multi-layer composite structure (Figure 1B). Their inner part is the cavity hosting the actual cell body (lumen), which in mature, specialized cells is surrounded firstly by a three-layered (S_1_, S_2_, and S_3_) secondary cell wall, then by a primary cell wall, and finally by a middle *lamella*, which separates neighboring cells.

This middle *lamella* has a variable thickness (from as thin as 0.2 µm to in excess of 1 µm, depending also on hydration), and its main (but not exclusive [35]) role as an intercellular ‘glue’ is due to it being predominantly made of (calcium-)gelled pectin. Proceeding inwardly, the thin (most commonly < 0.1 µm) primary walls feature rather disorganized cellulose microfibrils, with lignin as a minor component, and still significant amounts of pectin. Secondary walls are typical of cells having concluded their expansion phase; they are thick, up to 13 µm [36], and typically divided in layers, which differ in the orientation of cellulose microfibrils and in thickness, with S_2_ being the thickest (1–10 µm, 75–85% of the secondary wall thickness [36,37]). All three layers have cellulose as the major component (≈50%), followed by hemicelluloses and lignin in variable but comparable amounts, while pectin is typically absent (although phloem secondary walls may occasionally present it [38]). Of note, gymnosperm cells may further have a thin (<0.1 µm) “warty layer’’ on the innermost *lamella* of S_3_ [39]. Its name derives from the presence of wart-like protuberances, which are not responsible for the strengthening of the plant structure but affect permeability. Secondary cell walls are the largest part and most lignified cell wall compartment, owing to lignin rigidity and also hydrophobicity (hence barrier properties). Lignin can also be found in the middle *lamella* but in relatively small amounts and as non-interconnected aggregates [40], thereby not appreciably contributing to the mechanical properties of this layer. This 3D organization is summarized in Figure 1C. 

## 3. The Lignification Process: Building Blocks and (Bio)Chemistry

### 3.1. Monolignols (Lignin Building Blocks) from the Phenylpropanoid Pathway

**Figure 2:**
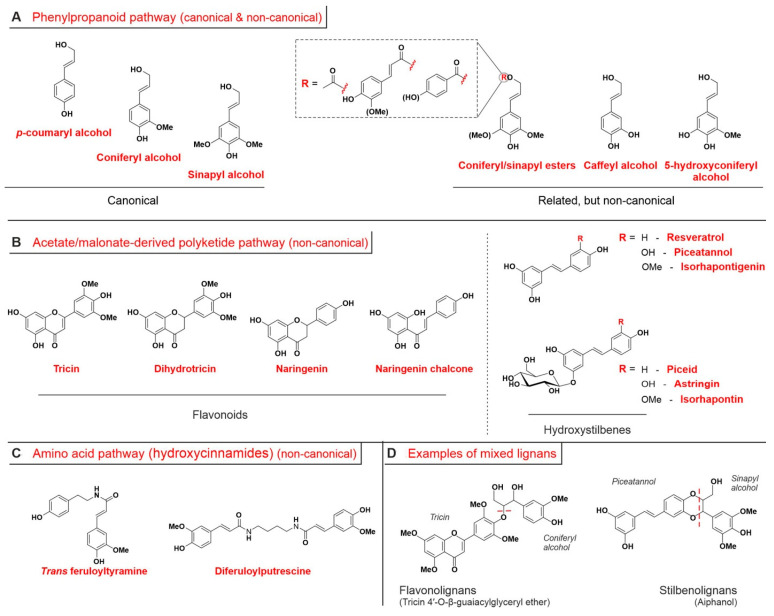
*(***A**). The three canonical monolignols (left: *p*-coumaryl, coniferyl and synapyl alcohol) are structurally very related to other lignols (right) produced through the same phenylpropanoid pathway (see Figure 3). Of note, the catechol groups in the caffeyl alcohol (producing C units in lignin) and 5-hydroxyconiferyl alcohol allow for a different radical/oxidative reactivity. (**B**). A few flavonoids (based on a polyphenolic α,β-unsaturated cyclic ketone structure (chromone); left) and hydroxystilbenes and their glycolides (1,3-diphenolic ring linked to a phenolic, catecholic or 2-methoxyphenolic ring through an ethylene residue; right) have been found to be monolignols too and are produced through the acetate/malonate polyketide pathway. Taxonomically, tricin is a flavon; dihydrotricin and naringenin are flavanones, and naringenin chalcone is—as suggested by the name—a chalcone. (**C**). Two hydroxycinammamides behave as monolignols. They are ferulic acid derivatives, which derive from the amino acid metabolic pathway. (**D**). Flavonolignans (left of the vertical dashed line) and stilbenolignans (right of the dashed line) are low MW products of reaction between a flavonoid (e.g., tricin) or a hydroxystilbene (e.g., piceatannol) and a phenylpropanoid lignol, whose sub-structures are separated by a red dashed line in the panel. While typical mechanisms for such reactions are listed in Figure 4. The reader is addressed elsewhere for more comprehensive lists of flavono- [63] and stilbenolignan [64] structures.

In this section, we discuss the biosynthesis of compounds that will eventually act as the lignin building blocks, i.e., the monolignols; please note that a detailed description of the corresponding units in lignin is to be found in Section 3.4 “Lignin units and relative lignin composition”. The most common set of lignin precursors is referred to as the canonical monolignols; these three C9 units are sinapyl alcohol (producing the so-called S units in lignin), coniferyl alcohol (G units), and *p*-coumaryl alcohol (H units), which is also known under the name of *p*-hydroxycinnamyl alcohol (Figure 2A, left). These monolignols have a common molecular motif, i.e., a phenol para-conjugated to a trans (E) double bond terminating in an hydroxymethylene groups, and they differ for the presence and number of methoxy groups flanking the phenolic OH. Although strictly speaking non-canonical, two structurally related classes of lignin precursors exist (Figure 2A, right): 

(1) γ-*O*-acylated compounds (esters), including acetates [41,42], p-coumarates (more common in grasses than woody plants) [42], and p-hydroxybenzoates [43]; 

(2) catechols such as caffeyl alcohol (yielding the so-called C units in lignin) or 5-hydroxyconiferyl alcohol (5HC units). Although structurally very similar to (methoxy)phenols, the presence of two neighboring OH groups produce a peculiar reactivity during lignification (see benzodioxane groups in Section 3.4) [44].

**Figure 3:**
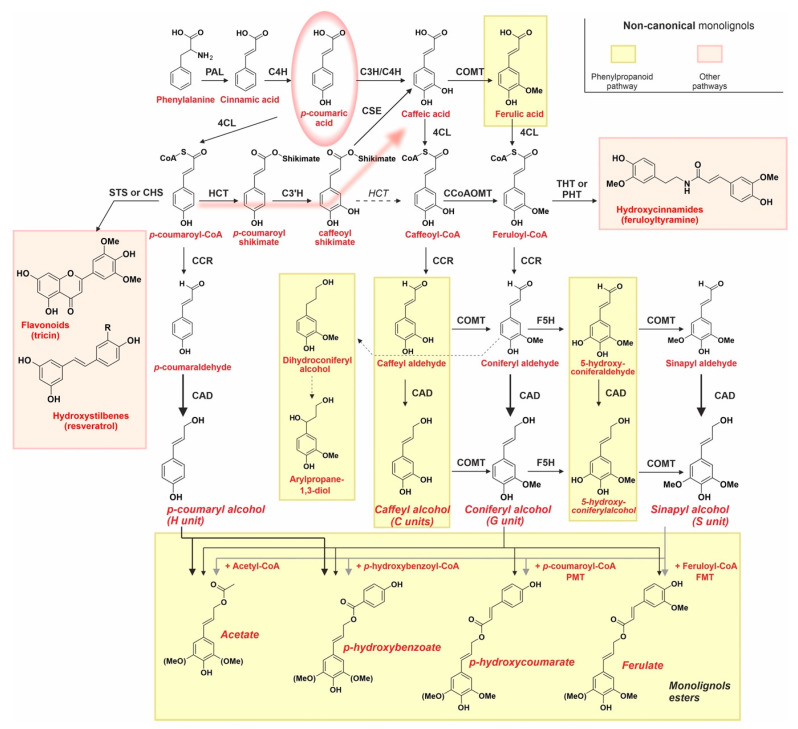
The phenylpropanoid pathway leads to the biosynthesis of both canonical (no background) and non-canonical monolignols (light yellow background), while other non-canonical monolignols are produced through different but connected pathways (pink background). Phenylalanine (Phe) is sequentially converted to *p*-coumaryl alcohol (H unit) by phenylalanine ammonia-lyase (PAL), cinnamate 4-hydroxylase (C4H), 4-coumarate:CoA ligase (4CL), cinnamoyl-CoA reductase (CCR), and cinnamyl alcohol dehydrogenase (CAD). *p*-coumaric acid (red circle) is the ‘hinge’ of all these biosynthetic paths, since not only it is the precursor of *p*-coumaryl alcohol, but directly (via *p*-coumarate 3-hydroxylase, C3H) or through *p*-coumaryl-CoA (the so-called shikimate shunt (blurred red arrow). Enzymes involved: *p*-hydroxycinnamoyl-CoA:quinate/shikimate (HCT), *p*-coumaroyl shikimate 3-hydrolase (C3′H), and caffeoyl shikimate esterase (CSE)) also lead to the production of caffeic acid and then to that of all non-canonical phenylpropanoid monolignols. There, multiple and redundant pathways lead to coniferyl (G unit) and sinapyl alcohols (S unit) and involve caffeic acid *O*-methyltransferase (COMT), caffeoyl-CoA *O*-methyltransferase (CCoAOMT), and ferulate 5-hydroxylase (F5H). Please note that among the non-canonical, phenylpropanoid-derived monolignols here we consider also compounds absent in Figure 2, such as ferulic acid (used in the formation of polysaccharide-lignin complexes, see later) or dihydroconiferyl alcohol, which is present in the lignin of CAD-deficient trees [65]. PMT: *p*-coumaroyl-CoA monolignol transferase (PMT). FMT: feruloyl-CoA with feruloyl-CoA monolignol transferase. For the non-phenylpropanoid pathway, hydroxystilbenes are produced through stilbene synthase (STS), and flavonoids are produced through chalcone synthase (CHS), whereas hydroxycinnamoyl-CoA:tyramine N-hydroxycinnamoyltransferase (THT) and hydroxycinnamoyl-CoA:putrescine hydroxycinnamoyltransferase (PHT), respectively, mediate the biosynthesis of diferuloylputrescine and of feruloyltyramine.

The structural similarities among the canonical and the above-described non-canonical lignols are due to a common biosynthetic route, which is referred to as phenylpropanoid pathway (Figure 3). The key intermediate of this process is *p*-coumaric acid, which is derived from phenylalanine (Phe) via the reductive deamination into cinnamic acid operated by phenylalanine ammonia lyase (PAL), followed by hydroxylation by cinnamate 4-hydroxylase (C4H). Unsurprisingly, a reduced expression of these enzymes leads to lower lignin production [45].

Another common point of all phenylpropanoid processes is that cinnamate carboxylates are always converted into primary alcohols by transforming them into coenzyme A (CoA) derivatives (through 4-coumarate: CoA ligase, 4CL) and by first reducing them to aldehydes (through cinnamoyl-CoA reductase, CCR) and then to alcohols (through cinnamyl alcohol dehydrogenase, CAD). Of note, the aldehydes are present in traces in most lignins, but they become particularly abundant in CAD-deficient plants [46], which tend to produce also some saturated non-canonical lignols (e.g., guaiacylpropane-1,3-diol) [47].

Other defining features of the phenylpropanoid pathway are as follows:

(A) The cytochrome P450 (CYP450)-dependent nature of hydroxylating enzymes [48], such as *p*-coumarate 3-hydroxylase (C3H, also known as coumaroyl shikimate 3-hydroxylase) [49] and ferulate 5-hydroxylase (F5H, also known as coniferyl hydrolase). Curiously, the downregulation of C3H and C4H genes in *Populus trichocarpa* increases the presence of lignol benzoate esters [50], which may be due to cross-talks between the benzoate and the phenylpropanoid pathways.

(B) The pervasive presence of 3- or 5-*O*-methylating enzymes, i.e., caffeic acid *O*-methyltransferase (COMT) or caffeoyl-CoA *O*-methyltransferase (CCoAOMT), which also show a certain degree of redundancy [51], since coniferyl (G) units can be produced by methylating caffeyl alcohol via COMT or caffeoyl-CoA via CCoAOMT. It is worth mentioning that 5HC units (in the second group of non-canonical lignols, i.e., catechols) are abundant in COMT-deficient plants [52,53,54]. A loss or reduction in COMT and/or CCoAOMT expression has also been invoked to explain C-lignin structure peculiar to cactus seed-coat, which is rich or uniquely composed of caffeic acids (C units) [55,56].

(C) A redundant biosynthesis of sinapyl alcohol, whose two paths see enzymes used in the sequence CAD-F5H-COMT-CAD or alternatively in the sequence F5H-COMT-CAD, i.e., producing first coniferyl alcohol or branching out at the level of its precursors aldehyde. In both cases, however, the production of sinapyl alcohol (leading to “S units” in lignin) requires that of coniferyl aldehyde (the precursor of the so-called “G unit”); this means that synapyl alcohol levels will not alter the quantity but the quality (the S/G ratio) of lignin. Indeed, the absence of F5H and COMT via selective mutations does not affect the overall amount of lignin but reduces the S/G ratio [57], which is conversely increased by F5H overexpression [58].

(D) A central role of caffeic acid. When its production is reduced by mutating caffeoyl shikimate esterase (CSE), the biosynthesis of all lignols except *p*-coumaryl is hampered, which means both a reduction in the total amount of lignin and its higher presence in lignin (“H units”) [59]. Of note, a certain degree of redundancy is present here too: caffeoyl-CoA can be produced directly from caffeolyl shikimate (through *p*-hydroxycinnamoyl-CoA:quinate/shikimate, HCT) [59], thereby bypassing caffeic acid.

(E) The common occurrence of γ-*O*-acylated ester conjugates (Figure 3, bottom). For example, acetates and *p*-coumarates make up to 80% of monolignols in (herbaceous) angiosperms such as *Hibiscus cannabinus* and *Agave sisalana* [42], while up to 45% acetylation has been found in (woody) gymnosperms such as *Carpinus betulus* [41]. Reportedly, the presence of esters is more common in the external layers of wood, e.g., the cork in *Quercus suber* [34]. Importantly, to our knowledge the enzymes responsible to the synthesis of these two esters have not been uncovered, whereas they have been identified for feruloylation [60] and p-hydroxycoumaration [61]. Last, there are diagnostic signatures for the presence of acetates, at least in the case of sinapyl derivatives: in their absence, β-β’ coupling of sinapyl alcohols produces bicyclic structures, the resinols (see later in Section 3.4 and Figure 6B), whereas in their presence the same reaction yields substituted tetrahydrofurans [62].

### 3.2. Monolignols from Other Pathways

Besides the three canonical and the several non-canonical monolignols derived from phenylpropanoid pathway (see previous section), in recent decades several other phenolic compounds have been recognized as true monolignols [66]. They are grouped in the categories of flavonoids and hydroxystilbenes (Figure 2B) and of hydroxycinnamic amides (Figure 2C); the former two are derived from coumaroyl-CoA [67,68], and the latter is derived from feruloyl-CoA [69] (pink boxes in Figure 3), thus by combining the phenylpropanoid pathway, respectively, with the acetate/malonate-derived polyketide (flavonoids and hydroxystilbenes) or the amino acid biosynthetic pathway (hydroxycinnamamides). Tricin [70] and resveratrol [71], either in their free form or in their many glycosides [72,73], are, respectively, the most commonly encountered flavonoid and hydroxystilbene in lignins.

Tricin was the first not directly phenylpropanoid-related compound to be recognized as a component of low MW flavonolignan structures (Figure 2D) [74] and then as an authentic monolignol. Tricin is almost ubiquitous in grasses or related plants, e.g., reeds such as *Arundo donax* [75], sugar cane [76], or *Cyperus papyrus* [77], bamboos such as *Phyllostachys pubescens* [78], and cereals such as *Sorghum bicolor* [79], where it typically concentrates in the aerial parts.

Piceatannol can be produced through processes unrelated to lignification; for example, in mammals it is the first metabolite of resveratrol [80]. Relatively recently it was also recognized as a lignol, and more specifically the first hydroxystilbene, during a careful analysis of the monomeric structures recovered from palm fruit endocarp lignin [71] after derivatization (conversion of alcohols to bromides with acetyl bromide) and reduction (elimination of bromide and phenol with acidic zinc dust to yield cinnamyl alcohols) [81]. Of note, and as it happens for flavonoids, several soluble low MW adducts hydroxystilbenes with lignols, i.e., stilbenolignans [82] such as aiphanol [83] (Figure 2D), have also been isolated.

Last, it is worth pointing out that while feruloyltyramine was recognized as a lignol almost 40 years ago, after being found in the *Nicotiana tabacum* lignin [84], the discovery of diferulyolputrescine as a lignol in maize kernels is considerably more recent [85]. 

### 3.3. Cell Wall Polysaccharides Involved in Lignification (Polysaccharidic Lignols)

Lignin would easily phase separate in cells walls, should it not be integrated with non-cellulosic polysaccharides, which include only some selected (feruloylated) members of the hemicellulose and pectin families.

**Hemicelluloses.** Hemicelluloses are typically seen as the internal ‘glue’ that binds together the structural elements of plant cell walls, i.e., cellulose microfibrils and lignin aggregates.

Hemicelluloses are a rather heterogeneous class of polysaccharides, common to wood and non-woody plants (up to 20–30% of the dry weight of the former and up to 40% in the latter [86]), which contribute to cell wall strength and rigidity through interactions with cellulose and lignin. In comparison to other plant-derived polysaccharides, e.g., cellulose, starch, pectins, and various gums, hemicelluloses have a significantly lower commercial value. However, they lack significant toxicity and are readily biodegradable, which has spurred some interest in applications such as food packaging [87] or biorefinery processes [88].

**Figure 4:**
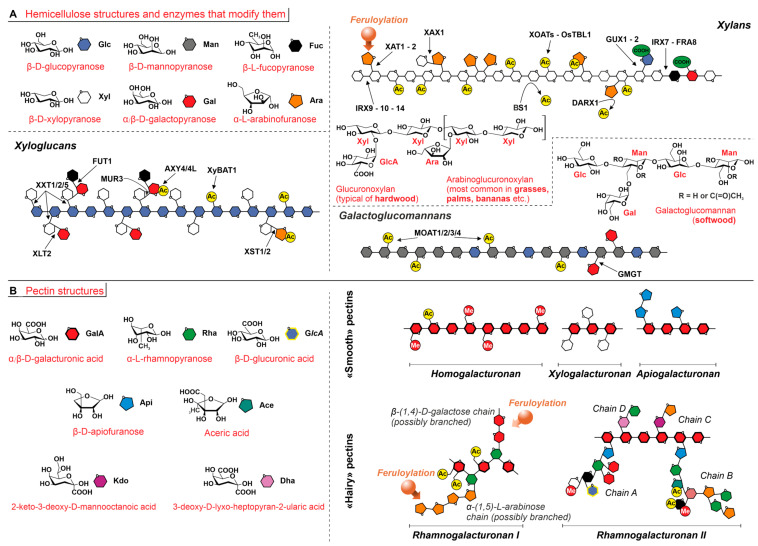
Hemicellulose and pectins; structures and symbols of the sugar monomers are reported in the left part of the panels; acetylated and methylated positions are highlighted with ‘Ac’ and ‘Me’ labels, feruloylation sites with orange arrows. (**A**). Most important classes of hemicelluloses and sites for the actions of some of the most common enzymes that modify them. In detail: in xylans, backbone biosynthesis is mediated by Irregular Xylem 9, 10, and 14 (IRX9-10-14), arabinosylation by Xylan Arabinosyl Transferases1 and 2 (XAT1-2), and further chain elongation with xylosyl residues onto arabinosyl side chains by Xylosyl Arabinosyl Substitution of Xylan1 (XAX1); finally, the addition of glucuronic acids is mediated by Glucuronic acid substitution of Xylan1 (GUX1). Other modifying enzymes include Acetyltransferases (XOATs and OsTBL1) and Acetylesterases (BS1 and DARX1) that introduce and remove acetyl groups. In xyloglucans, xylosyl residues are added onto the glucan chain through Xyloglucan Xylosyl Transferase 1, 2, and 5 (XXT1-2-5, respectively, for isolated xylosyl residues, or those condensed with a galactosyl or with galactosyl-fucosyl units), are added onto Xyl-Gal dimeric side chains through Xyloglucan L-side chain galactosyl Transferase position 2 and through Murus3 (XLT2 or MUR3), and are added onto Gal-Fuc through FUcosyl Transferase 1 (FUT1). The formation of Xyl-Araf side chains is mediated by arabinoSylTransferases 1 and 2 (XST1-2), and the acetylation pattern is, respectively, regulated by Xyloglucan *O*-acetyltransferase 1 (AXY4-4L) on fucosylated galactosyl and by Xyloglucan Backbone *O*-Acetyl Transferase 1 (XyBAT1) on backbone glucosyl residues. In galactoglucomannans, the introduction of galactosyl side chains is mediated by GalactoMannan GalactosylTransferase (GMGT), and the acetylation pattern mannan is mediated by *O*-acetyltransferases (MOAT1-2-3-4). (**B**). “Smooth” pectins typically have a partially methylated, linear homogalacturonan (HG) backbone. If HG chains feature short branches in the form of β-(1,3) and β-(1,2)-linked D-xylose or β-(1,5)-linked D-apiose residues, they are respectively referred to as xylogalacturonan (XGA) and apiogalacturonan (AP). “Hairy” pectins are heavily branched macromolecules. Rhamnogalacturonan I (RG-I) has alternating (acetylated) GalA and α-(1,2)-rhamnosyl residues, the latter bearing oligo(galactose) and oligo(arabinose) branches, where feruloyl residues may be present. Rhamnogalacturonan II (RG-II) typically features a short (7–9 units) HG main chain with a large variety of branches (four types of side chains, named A to D) made up of up to 12 different saccharides including uncommon monomers, e.g., β-D-apiofuranose (Api), aceric acid (Ace), 2-keto-3-deoxy-D-mannooctanoic acid (Kdo), and 3-deoxy-D-lyxo-heptopyran-2-ularic acid (Dha). Chains C and D are dimeric (Dha-Rha and Kdo-Ara), whereas chains A and B show a large architectural diversity.

Structurally, all hemicelluloses share the same glycosidic bond configuration β-(1,4) of cellulose but are shorter (from about 100 in softwood to up to 200 in hardwood) [89] and above all largely vary in composition (Figure 4A) and architecture, whereas cellulose is strictly a linear homopolymer of glucose (Glc). Hemicelluloses, on the contrary, not only also include mannose (Man) and xylose (Xyl) repeating units in their main chain but also feature side chains (=they are branched), which are based on uronic units (mostly α-D-glucuronic acid, possibly methylated) and irregular patterns of α-L-arabinofuranose (Araf), α-D-xylopyranose (Xylp), α-galactopyranose (Galp), or α-L-fucopyranose (Fucp) units as side chains. Depending, therefore, on their composition, polysaccharides categorized as “hemicelluloses” are more properly termed xylans, xyloglucans, and galactoglucomannans (all depicted in Figure 4A), as well as glucomannans, mannans, and β-(1,3:1,4)-glucans. We here do not consider other similar cell wall polysaccharides, e.g., galactans, arabinans, or arabinogalactans, as hemicelluloses, because they may participate in pectin biosynthesis and/or because of the different glycosidic configurations.

Xyloglucans are mainly present in primary cell walls and make up about 20 wt% of these structures in hardwoods, around 10 wt% in softwoods [90]; due to the limited thickness of the primary walls, however, xyloglucans are generally not the major components of the hemicellulose pool. The most common hemicelluloses in secondary walls are galactoglucomannans in softwoods and xylans in hardwoods; for example, 20 wt% of the hemicellulose is composed of *O*-acetyl-4-*O*-methylglucuronoxylan (a xylan) in birch (hardwood) but of *O*-acetyl-galactoglucomannan in spruce (softwood) [91]. Last, it is also worth pointing out that while glucuronoxylans are major constituents of hardwood hemicelluloses, those in grasses (and commelinids such as bananas, gingers, palms, etc.) are predominantly made of the structurally similar arabinoglucuronoxylans [90].

It is noteworthy that, in addition to the differences in the sugar residues of their backbones and side chains, another source of hemicellulose heterogeneity comes from their continuous modification by a variety of enzymes (main enzymes and their modification sites depicted in Figure 4A). For example, the acetylation degree of xylans (high: they account for the vast majority of cell wall acetyl esters [92]) is inversely proportional to their hydrogen bonding capabilities and therefore to their interactions with cellulose. Among the other forms of side chain derivatization, it is worth mentioning the introduction of methoxyphenol groups, typically in the form of ferulic esters *O*-2 [93] and *O*-5 [94] linked to arabinose residues in the side chains of xylans [95]; such residues can oxidatively dimerize and/or react through a variety of coupling geometries, which will be discussed in more detail in the next section.

In summary, the heterogeneity of hemicelluloses is not only due to the synthetic modalities of their backbone but also to their enzymatic post-processing, which also provides the capacity for them to interact with cellulose and lignin.

**Pectins.** Pectins (pectic polysaccharides) are the major component of the middle *lamella* but are also abundant in primary walls; both this localization and the timing of their deposition (early growth phases) would appear to make them almost mutually exclusive with lignin, which is predominantly localized in secondary walls and is produced after the cells have reached their final dimensions.

The main building block of pectins is α-(1,4)-linked D-galacturonic acid (GalA), which is often acetylated or methylated and combined with a variety of comonomers, such as α- and β- anomers of L-Fucp, L-Araf, L-Rhap, D-Manp, and D-Xylp (refer to Figure 4B for full names and structures). The following pectin structures typically exist as independently, but in principle are also potentially present as separate domains of larger macromolecular structures: 

(A) Unbranched homogalacturonans (HG), often referred to as “smooth” pectins. HGs have a linear backbone with about 100 α-(1,4)-linked GalA units, which can be methylated and/or acetylated. Of note, HG may be produced in a highly methylated form and then demethylated in a random or blocky fashion, with the former organization being useful to reduce ‘stickiness’, e.g., during cell division [96], and the latter being on the contrary capable of calcium-mediated gelation. Two rather recently discovered derivatized forms of poly(galacturonic acid) are xylogalacturonan (XGA), which is predominantly found in leaves [97], and apiogalacturonan (AP), which has been found in aquatic plants such as duckweeds [98] and seagrasses [99].

(B) Rhamnogalacturonans I (RG-I). [α-D-GalA-(1,2)-α-L-Rha-(1,4)-] dimeric units form GalA/Rha alternating polymers, which are typically acetylated at GalA, and C-4 branched with linear or branched oligo(α-(1,5)-L-arabinose) and oligo(β-(1,4)-D-galactose).

(C) Rhamnogalacturonans II (RG-II) [100]. RG-IIs are low MW (5–10 KDa) and highly branched polymers of more than twelve different sugars. The main chain is made of GalA residues with a low degree of methylation or acetylation, while four different types of side chains exist, all with a complex composition (A to D in Figure 4B). A and B are, respectively, 7- and 6- to 9-residue-long, branched chains comprising at least five different sugar types. C and D are disaccharides composed of peculiar sugar residues (e.g., 3-deoxy-lyxo-2-heptulosaric acid (DHA) and 3-deoxy-manno-2-octulosonic acid (KDO)).

In terms of the relative ratio between the various components, this is very variable. Most sources report 60–65% of pectin being generally made up of HG [101], and this may also comprise the 6–7% of XGA recorded in leaves [97] or large amounts of AP in some aquatic plants (where it may even replace HG, whereas in others is possibly replaced by XGA [102]. Of note, these data may also be affected by the sample treatment for sugar analysis: for example, AP may be under-represented due to its recalcitrance to pectinases [103]. However, general pectin compositional data should be taken with a pinch of salt, and these “smooth” pectin components may not always account for the majority of pectin: for example, the content of RG I has been measured from as low as 11% (in Arabidopsis [104]) to up to 85% (in Okra pods [105]) of the total pectin content (itself very variable). Further, since RG-I is also associated with water-holding capacity and firmness of plant tissues and its degradation to fruit maturation processes [106], its content would further vary throughout the plant life cycle.

Pectins associate intermolecularly to the point of producing gels; mechanistically, the association proceeds through (1) Ca^2+^ bridges, in HGs (provided that sufficiently long demethylated sequences are available [107]); (2) formation of borate-diol esters, which are most typical for RG-II (e.g., causing its dimerization [108,109] and contributing significantly to cell wall modulus [108,109]) but have been shown to be operational also in the formation of mixed HG/RG-I/RG-II aggregates [110]; and (3) oxidative coupling reactions involving the side chains. Feruloyl esters are indeed present in pectins, where they are linked to arabinan and galactan sequences, i.e., the side chains of RG-I [111,112], with an overall content that can be as high as the 0.8 wt% of pectin [113].

**Feruloylation as a route to hemicellulose and pectin integration with lignin.** Both hemicelluloses and pectins can therefore bear ferulate side chains [114]: the presence of ferulic acid polysaccharide esters in cell walls is known since the ‘70s [115], and the first reports of arabinose and galactose-linked ferulates (hinting therefore to xylans–and specifically arabinose-containing ones-and RG-I as the carrier components) go back to the early 1980s [116]. Ferulates are well known to undergo oxidative dimerization [115], which can already cross-link the polysaccharides they are part of [117], and both monomeric and dimeric ferulates can undergo further oligomerization [43,118,119]. These oxidative processes are well-known to strengthen hemicellulose networks and allow them to covalently bridge to lignin through the formation of polysaccharide-lignin complexes [120,121,122] and thereby also to act as a template for lignin deposition [123] and to decrease the degradability of cell walls [119,124]. The occurrence of other covalent interactions in these complexes, such as reactions on p-coumarates and transesterification reactions [125], cannot be discounted. Indeed, p-coumarates possibly play a role similar to ferulates (e.g., dimerizing) [116,126] but are less studied due to their lower amounts. It is also worth pointing out that ferulate-based cross-linked structures are often seen as a marker of the switch between the deposition of primary cell wall (relatively poorer in ferulate) and that of the secondary one [127]. Since the latter is essentially devoid of pectin, this may be taken as an (erroneous) indication of a generally poor pectin–lignin integration, which would tally with their opposite concentration profile (see Figure 1C). However, lignification actually starts from the pectin-rich areas of middle lamella and cell corners [40], and feruloylated cell walls have often been suggested as potential nucleation sites [114], along with tricin (tricin deficiency leads to poor lignification [128]), which may actually mean that pectin integrates to an initial and less aggregated form of lignin.

### 3.4. Lignification (Bio)Chemistry

**Figure 5:**
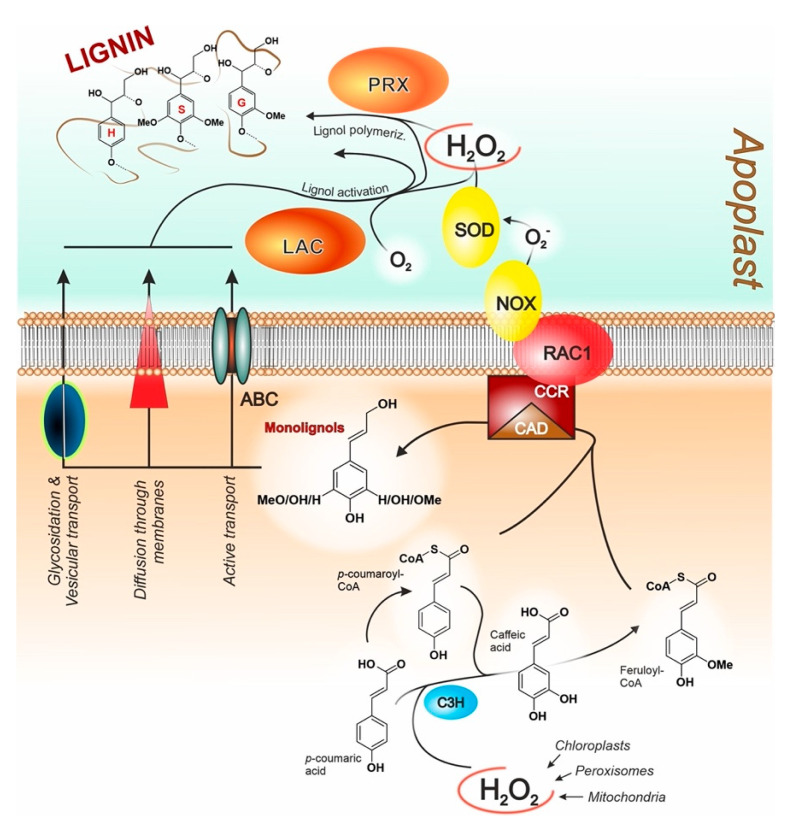
The biosynthesis of monolignols, the formation of lignin via enzymatic oxidative coupling, and the hydrogen peroxide (H_2_O_2_) detoxification system are summarized by using canonical structures, although the scheme is valid in principle for most if not all monolignols. Please note that the extracellular space, here generically defined as the apoplast, is the site for the production not only of lignin, but also suberin, cutin, and other biopolymers. Acronyms: C3H—*p*-coumarate 3-hydroxylase (acting also as a cytosolic ascorbate peroxidase and therefore also referred to as C3H-APX); CCR—cinnamoyl-CoA reductase; CAD—cinnamyl alcohol dehydrogenase; ABC—ATP-binding cassette transporter; RAC1—the Rho GTPase Ras-related C3 botulinum toxin substrate 1; NOX—NADPH oxidases, also known as respiratory burst oxidative homologs (RBOHs); SOD—superoxide dismutase; LAC—laccases; PRX—class III peroxidases (Class I: microbial or intracellular plant peroxidases. Class II: extracellular fungal peroxidases. Class III: extracellular plant peroxidases).

This discussion initially focuses on the biochemical/biophysical environment of lignification (Figure 5); for the sake of simplicity, here we only discusss phenylpropanoid monolignols but identical concepts can be applied to other lignols too.

**A short summary of the processes.** Intracellularly, most phenylpropanoid monolignols have a very similar biosynthesis: they have a common precursor (p-coumaric acid, see Figure 3) and a common intermediate (caffeic acid, the path leading to p-coumaryl alcohol being the exception). At the end of their biosynthesis, monolignols are moved across the cell membrane into the extracellular space (the apoplast) through a variety of mechanisms [129]: (1) passive diffusion, if they are sufficiently hydrophobic to solubilize in the membrane [130]; (2) vesicular transport, which has been shown to be operational for the very hydrophilic glycosylated lignols [131]; (3) active transport through membrane transporters [132], although - to our knowledge - to date only one transporter has been identified, which selectively acts on *p*-coumaryl alcohol [133]; and (4) transport through channels, which have been postulated [129] but to our knowledge not experimentally verified yet (thus not shown in Figure 5). Extracellularly, under the assistance of laccases and peroxidases, monolignols are activated, typically producing radicals at phenol OH groups, and then polymerize oxidatively. Of note, the very same enzyme classes are also those capable of lignin degradation [134,135], always through oxidative mechanisms.

**Hydrogen peroxide plays a key role throughout the lignification process.** H_2_O_2_ fulfills a variety of roles in plants [136], but it is specifically pivotal during lignification. Intracellularly, the biosynthetic routes to monolignols are mostly based on the cytosolic C3H enzyme, which is not only a 3-hydrolase but also an ascorbate peroxidase and is hydrogen peroxide-dependent [137]. Also CSE, i.e., the key enzyme of the so-called shikimate shunt to caffeic acid, is hydrogen peroxide-dependent [138]. Extracellularly, all peroxidases require H_2_O_2_ as a co-factor [139]; NADPH Oxidases (NOX) are a major source of this extracellular hydrogen peroxide (through superoxide dismutation by SuperOxide Dismutase, SOD). Of note, NOX have been reported to be activated in a RAC1-mediated fashion by CCR [140], which on its turn reportedly can associate to CAD in a single, membrane-localized complex [138] that converts monolignols’ CoA thioesters into primary alcohols, thereby providing a putative link between intra- and extracellular processes. Another important source of apoplastic (=extracellular) H_2_O_2_ are indeed laccases, which produce it during lignol activation (generation of phenol radicals). Also in this case, the liberation of hydrogen peroxide usable in lignol polymerization is therefore tied to events with an upstream position in the lignification chain.

**The enzymes: laccases and peroxidases**. The polymerization of lignols is the final phase of the lignification process. It is assisted by two classes of enzymes, laccases (multi-copper oxidases, which depend on molecular oxygen) and peroxidases (iron-heme oxidases, which employ hydrogen peroxide), both capable of catalyzing/assisting a wide variety of oxidation reactions. Their significance for plants is witnessed by their number and level of expression: in *Arabidopsis thaliana*, 17 laccase [141] and 73 class III peroxidase genes [142] (several linked to lignification [143]) have been found; among them, LAC4 laccase and PRX64 peroxidase (the latter involved in the build-up of the Arabidopsis Casparian strip [144]) are the most highly expressed oxidative genes in that plant. Interestingly, the expression of these two enzymes is topologically different, with the former preferentially located in secondary cell walls, the latter confined to the middle lamella and cell corners [145], but this is not a general feature: a peroxidase such as PRX72 is localized in cell walls, and LAC4 itself is transiently expressed in cell corners, and there are also laccases and peroxidases that localize in non-lignified tissues [146].

The first step in the chain of reactions leading to polymerized lignin is the introduction of free radicals in monolignols, i.e., their activation, typically at the phenol OH. Evidence from *Arabidopsis* shows that lignols are activated by laccases and not by peroxidases [147]. The latter are heavily involved in lignol oligo/polymerization, although laccases are involved in this phase too: in *Arabidopsis*, double mutants deficient in LAC4 and LAC17 have shown hypolignified fibers and collapsed xylem vessels [148], and the additional loss of function of LAC11 has led to growth defects and failure [147]. Furthermore, also single or double mutants in several peroxidase genes have shown reduced lignin, even if typically not as much as in laccase mutants; for instance, the prx72 mutant results in a lignin content reduction up to 35% [149].

**Figure 6:**
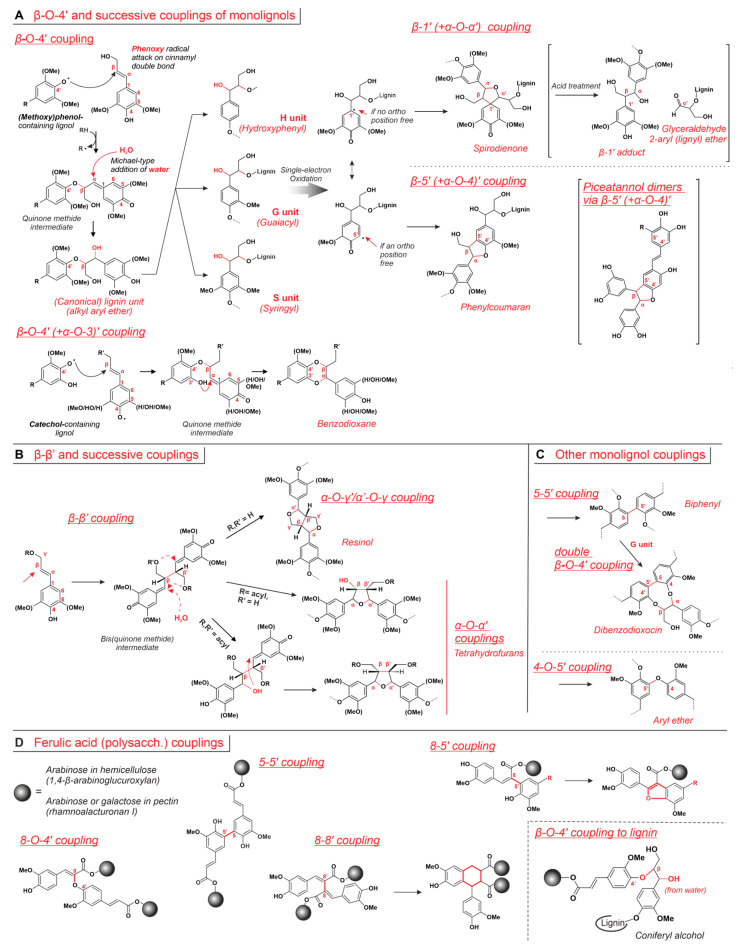
All new bonds formed during lignol couplings, and the numbering of all relevant atoms are shown in red. (**A**). Reaction paths and structures of lignin units deriving from β-*O*-4′ coupling. Please note that G (guaiacyl) units are structurally related to guaiacol but actually are derived from coniferyl alcohol, and “S” stands for “syringyl” although the corresponding monolignol is sinapyl alcohol. (**B**). β-β’ couplings produce bis(quinode methide) intermediates, which may react with intramolecular alcohols and/or water, producing bicylic resinols or various tetrahydrofurans. The stereochemistry of these products is typically ascribed to the action of dirigent proteins [157]. (**C**). Two relatively less common coupling mechanisms (5-5′ and 4-*O*-5′) lead to dibenzodioxocin- and diaryl ether-containing junctions. Please note that 5-5′ can join two G units but is operational also in linking together a G and an H unit [171]. (**D**). Examples of coupling reactions involving ferulic esters [117], which also account for ferulate dimerization and conjugation to lignin of hemicellulose/pectin. Of note, the β carbon of canonical monolignols is referred as the 8 carbon in ferulates.

In short, it is widely accepted that both laccases and peroxidases participate in the polymerization phase; however, the level of involvement of these (or other) enzymes has been long debated, with the two options of them directly participating in the process or having a more distant, assistive role. As nicely summarized in a review by Ralph et al. [150], the current consensus opinion is that lignol activation/polymerization processes are largely combinatorial, chemically controlled processes; i.e., the large number of different monomers (up to 35 [151]) react in a fashion that is predominantly dependent on their molecular accessibility reactivity, absolute concentrations and relative stoichiometric ratios, their supply rate [152], and the local pH [153], as much as the enzyme concentration and activity [154], rather than specific interactions with the active sites of the involved enzymes. This tallies with the fact that peroxidases may operate on lignols not only by direct catalysis reactions on monolignols but also through hydroxy radicals that they are known to generate [155]. There is, however, evidence of protein-driven direct assistance, although not mediated by laccases or peroxidases; for example, these enzymes in vitro provide racemic resinols via β-β’ coupling (see Figure 6B), whereas in vivo the same reaction products are optically active [156]. This has led to the discovery of so-called dirigent proteins [157,158,159,160], which at least for this specific reaction orientate the stereochemistry of the product, although not being directly capable of chemical catalysis.

Finally, it is noteworthy that lignin degradation (e.g., by fungi) is based on laccases and peroxidases too. From the first finding of a peroxidase capable of lignin degradation [161] and that other oxidases (laccases) shared this capacity [162,163], it has gradually become clear that laccase may have possibly a greater role [164], but peroxidase has a wider variety (lignin peroxidases, manganese peroxidases, and versatile peroxidases) [134].

**Chemical reactions of lignol oligo/polymerization.** In the early stage of lignification, monolignol units dimerize to form dilignols; in this phase, β-*O*-4′ coupling leads to alkyl aryl ethers, e.g., the canonical lignin units, that may later undergo β-5′ reactions to phenylcoumarans or β-1′ reactions to spirodienones (all in Figure 6A). Another common coupling mechanism is the β-β’, leading to resinols (Figure 6B). It is noteworthy that 4′-*O*-β is also the typical coupling mechanism detected for non-canonical tricin, and the apparent lack of homoligomers [70] and its preferential presence in low MW lignins [165] suggest tricin to possibly act as a nucleation site [166].

The general mechanism for the β-*O*-4′ coupling (Figure 6A, top) sees first the attack of a phenoxy radical to the β atom of a lignol; this leads to a quinone methide, which is a strong Michael-type acceptor and rapidly undergoes a second reaction with a nucleophile. When the latter is a water molecule (in red), the canonical H, G and S lignin structures are produced.

The canonical H, G, and S units produced via 4′-*O*-β coupling can then undergo further reactions: if the phenol OH is free in the ortho position, the radical attacks onto other lignols’ double bonds, and the subsequent β-5′ ring closure produce phenylcoumarans. Of note, the same kind of reaction is also used to yield some piceatannol dimers [83] (in brackets in Figure 6A, middle right). Should such positions not be available, e.g., in S units, the ring closure employs an aliphatic alcohol, thereby generating spirodienones due to hindered rearomatization. Of note, these units were discovered only rather recently [167], because their lability during lignin extraction processes led to the isolation only of their 1,2-diarylpropan-1,3-diol degradation products [168] (see Figure 6A top right).

A different reaction output would occur if in the original β-*O*-4′ coupling the attacking phenol was actually a catechol (Figure 6A, bottom), e.g., a non-canonical monolignol, such as 5-hydroconiferyl alcohol, piceatannol, or astringin, or catechol-containing lignin units (aka C units)). The quinone methide would not react with water but with the neighboring OH group, producing benzodioxanes, which indeed have been first found in COMT-deficient and therefore 5HC (catechol)-rich plants [52,169]. Of note, a number of piceatannol-based benzodioxane found in alcoholic extracts of the grass *Cyperus longus* are produced following this mechanism [170].

Another important class of reactions is the β-β’ dimerization, leading to bicylic pinoresinol or monocylic tetrahydrofuran structures previously mentioned in relation to the stereochemical assistance by provided by the so-called dirigent proteins [156,158]. This pathway always starts through the β-β’combination of two coniferyl or sinapyl radicals, leading to a bis(quinone methide); it is worth noting that, besides Michael-type addition of either water or intramolecular alcohols (as in Figure 6B), this intermediate has also been found to potentially undergo a reduction and produce its saturated analog secoisolaricinol [172].

Other noticeable lignol reactions are the 5,5′ and 4-*O*-5′ couplings (Figure 6C), which produce groups (respectively, biphenyls and diaryl ethers) particularly resistant to enzymatic degradation, e.g., via β-etherase [173] or pyrolysis [174]. Although diaryl ethers are likely easier to dissociate than biphenyls, they still have much higher dissociation energies than all other alkyl aryl ethers [175]. Since 5-5′ coupling requires at least one ortho position to phenolic OH to be free, this reaction is common in woods with a low content of S units (softwoods), where it occurs on G units [176] or also on ferulates [177]. If the 5-5′ biphenyls bear non-etherified phenols, they may further react with monolignols and produce 8-membered cyclic structures referred to as dibenzodioxicins [178,179,180].

Finally, it is worth mentioning that ferulates undergo essentially the same kind of reactions (Figure 6C), with the only significant difference being the initially formed structures are predominantly dhydrodimers; i.e., they still contain cinnamoyl double bonds (i.e., conjugated to both phenyl and carboxylate groups) [117,181], whereas in most other lignol dimers this unsaturation is lost. This is likely due to the different reactivity of the quinone methides, which appear to favor a rearrangement that recovers the conjugation to ester carboxylates rather than Michael-type addition.

**Lignin units and relative lignin composition.** Firstly, a warning: lignin compositional data should be used with considerable caution, due to possible biases of both isolation and analytical procedures. For example, isolation of lignin through thioacidolysis from milled wood has shown to very considerably reduce the level of phenol etherification [182], whereas the presence of 4-*O*-5′ products may have been historically underestimated, due to their difficult analysis via NMR [183,184], and the same applies to β-1′ spierodienones due to their above-mentioned acid lability [168].

Having said this and further adding that the specific make-up of lignin units may depend not only on the plant species but also on the environmental conditions experienced by the individual plant, a consolidated point is that β-*O*-4′ alkyl aryl ethers are by far the most common products encountered in a vast range of lignins. For example, in grasses and grains they range from being present in 45–49% of the units in bamboo (β-β’ resinols 3.6–7.4%, β-β’ tetrahydrofurans 2.0–2.3%, β-5′ phenylcoumarans 2.8–4.5%, β-1′ spirodienones 1.3–2.3%, and 4-*O*-5′ diaryl ethers 2.8–2.9%) [78], through 58% in flax fibers (β-5′ phenylcoumarans 11%, β-β’ resinols 9%, and lower amounts of spirodienones and dibenzodioxocins) [185], 72% in jute (β-β’resinol 16%, β-5′ phenylcoumarans 4%, and β-1′ spirodienones 4%) [186], 75% in straw (β-5′ phenylcoumarans 15% and 5-5′/β-*O*-4′ dibenzodioxocins 3%) [187], and 77–79% in spent grain from brewing (β-5′ phenylcoumarans 11–13%, β-β’ resinols 5–6%, and 5-5′/β-*O*-4′ dibenzodioxocins 3–5%) [188], to up to 82% in elephant grass [189] and 83% in sugarcane (β-5′ phenylcoumarans 6%) [187]. Also, in woody plants β-*O*-4′ alkyl aryl ethers have a similarly dominant position: for example, they account for 46–50% of lignin units in *Eucalyptus globulus* (β-β’ resinols 10–14%) [190] and 68–77% in the cork oak *Quercus suber* [34] in hardwoods and from 40 up to 50% in various softwoods [191,192]. Of note, while 5,5′ biphenyls are the second most common linkages in softwood (up to 26% of the linkages, with an average of 10% for the third most common β-5′ phenylcoumarans), they are comparatively much less common in hardwood (up to 9%) [192].

It has been suggested that β-*O*-4′ couplings are more common in the early stages of lignification, and reportedly this may not be due to them being favored in the initial production of dilignols (β-5′ and, to a lesser extent, β-β’ may be favored) but to β-*O*-4′ being preferred for the chain extending reaction of monolignols with di- and monolignols [192]. Equally interestingly, under conditions of limited supply of monolignols, e.g., because of their difficult diffusion in an already lignified region, the dominant reactions may be 5-5′ and, less, 4-*O*-5′ cross-linking between lignin oligomers [192], which explains why the corresponding units are quantitatively etherified [182] (i.e., the reaction should not preferentially involve monolignols, which have free phenol groups). This would mean a lower content of β-*O*-4′ in older, more lignified plants or in the tissues more distant from their centers (cork being most lignified, xylem least). Indeed, in the cork oak *Quercus suber*, the amount of β-*O*-4′ units has been found to decrease radially from 77% in xylem, through 71% in phloem, down to 68% in cork, and in the latter condensed structures are most abundant (β-5′ phenylcoumarans 20%, 5-5′/β-*O*-4′ dibenzodioxocins 5%) [34]. Further, in *Eucalyptus globulus* β-*O*-4′ units have also been found to slightly decrease with age (and β-β’ resinols marginally increase) [190]. These features, however, may be more common in woody plants than in the less lignified grasses; indeed, in, e.g., elephant grass (as the name suggests, a grass), pith and cortex appear to be similar in composition [189].

In terms of the structural details of the units produced via β-*O*-4′ coupling, it is noteworthy that:

(A) H units are by far the least common, typically between 0.5 and 10% of total lignin [151], although in some grasses they may reach up to >30% [193]. 

(B) G units are particularly frequent in softwood; for example, spruce lignin can have up to 99% of G units [194], and similar values are seen also in non-woody plants such as the Musa textilis banana [195].

(C) Hardwoods have a more variable S/G ratio, typically between 1:4 and 4:1; for example, the ratio is 1.2:1 throughout heartwood, sapwood, and bark of teak (with H units increasing, respectively, from 2 to 5%) [196], ≈3.5:1 in the heartwood and sapwood of *Eucalyptus globulus* [197], 1.4:1 in magnolia, 2.5:1 in birch, and 3.3:1 in beech [198]. There are also reports of significant variations within the body of a wood plant; for example, the S/G ratio varies from 1.2:1 in the heartwood and sapwood, through 1:1.4 in the phloem to 1:6.5 in the bark of the cork oak [34]. Importantly, however, this does not imply that wherever lignin has significant amount of S units, the younger the lignin, the higher is its S content: for example, in grasses such as tall fescue, the S/G ratio tends to decrease (higher S content) during stem development [199].

A final note refers to the couplings involving ferulates, since they may significantly depend on the structural details of their lignols: 80% of feruloyltyramine undergoes β-*O*-4′ and β-5′ (couplings), with the remaining 20% being attached to cell walls through their phenolic moiety [200], while diferuloylputrescine is almost exclusively incorporated via β-5′ linkages [85].

**Targeted alterations to lignification.** Lignin has traditionally been seen as an obstacle for the valorization of plant biomasses, e.g., reducing their degradability and therefore providing a less efficient energy source in animal foraging. However, this general view does not take into account that lignin composition is a major determinant of this: for example, with appropriate extraction/treatment processes, higher S/G ratios are linked to, e.g., much increased enzymatic degradability of bamboo residues [201], easier digestibility of roughage from various sources in ruminants [202], or better methane production following anaerobic digestion of birch biomass [203,204]. Therefore, in several studies it has been tried to modify the lignification process in order to reduce the overall lignin content or to alter its composition, in order to improve its later properties.

Hereafter, we provide examples of three biotechnological strategies employed to achieve these targets (lignin reduction or lignin modification), but firstly it is important to stress that the overall amount of lignin appears not to be really critical for plant development; for example, when in Arabidopsis, Thaliana genes involved in lignol production (e.g., C4H, 4CL, CCoAOMT, CCR, and CAD; see Figure 3 for an overview of their roles) were individually mutated, lignin production decreased, with large increases in that of hemicelluloses and no significant effect on that of cellulose but no major effect on plant growth [205].

(1) Alteration of lignol biosynthesis. This approach utilizes the lignin-modifying effects of the reduced expression/activity of enzymes involved in lignol production. Possibly the best example is offered by CAD, whose decreased activity impairs the reduction of all aldehydes to canonical monolignols and thereby greatly increases the aldehyde lignin content [206,207]. A lower CAD activity appears to have a disproportionate effect on S units, as shown in CAD-mutated *Arabidopsis* (S/G ratio decreased from 1.5:1 to 1:2 [205], linked to much increased degradability [206]) or in alfalfa treated with anti-CAD antisense (S/G ratio decreased from 1:1 to 1:2–3) [208], which is likely ascribed to higher specificity of these enzymes for sinapylaldehyde [209]. These findings fit with the observations that naturally occurring CAD mutant plants (whose lignin is particularly rich in aldehydes but appear otherwise rather normal [65,210,211]) appear to have increased digestibility, as shown for the CAD-mutant varieties of Sorghum bicolor [212], and may be the case for the specifically silkworm-friendly Sekizaisou mulberry trees [213]. Of note, some other Sorghum bicolor varieties are natural COMT mutants [214], as much as some varieties of poplar [215]. The better degradability of their biomass [216] is also in this case associated with a lower S/G ratio, in addition to a much lower overall lignin content.

Another important example is offered by the hydroxylating enzyme F5H, which is a key player in the production of synapyl alcohol. In rice, its downregulation enriched lignin in G units (thereby making it in principle more degradable), while its upregulation does the contrary [217]. In poplar genetically engineered to express an *Arabidopsis* F5H, the resulting higher-S lignin showed an increased resistance to wood-decaying fungal attacks [218] (although in other cases, such as the resistance of *Brassica napus* to *Sclerotinia sclerotiorum*, it may be associated with a higher G content produced by F5H knockout [219]). Of note, while reduced F5H and COMT both have the potential to reduce the S/G ratio (comparatively seen, e.g., in sugarcane [220]), when an F5H overexpression was induced in the presence of a nonsense mutation of COMT, the former did not compensate for the latter, and the final lignin was still low in S units [221].

Besides changing the S/G ratio, lignol synthetic enzymes can also affect other descriptors of lignin composition. For example, the downregulation of the caffeic acid-producing C3H has led to a lignin abnormally high (up to 65%) in H units; this was accompanied by the apparent absence of β-1′ coupling products, since their ortho reactivity, e.g., via β-5′ coupling, is favored for coumaryl alcohol-derived units [222].

Last, it is worth mentioning that, by silencing the flavonoid-producing CHS, a lignin with highly reduced tricin was obtained, whose higher density of β-β’ and β-5′ units indicated a higher likelihood of monolignol dimerization and therefore supported the hypothesis that tricin predominantly acts as an initiator group [223].

(2) Alteration of lignol transport. Knockout mutants lacking the only confirmed monolignol transporter (AtABCG29, reportedly a selective exporter *p*-coumaryl alcohol and therefore in principle affecting only H units) showed a 25–30% lower content in all lignin units, which the authors of the study interpret mainly as a consequence of cross-talks between the phenylpropanoid pathways [133] but may also be the result of the transporter acting also on p-coumaroyl-CoA, the general precursors of all canonical lignols (Figure 3).

(3) Alteration of lignol polymerization. Peroxidases are a positive determinant of the deposition of lignin in cell walls; for example, in tobacco plants, the introduction of additional peroxidase genes has been shown to both significantly enhance hydrogen peroxide production and increase the lignin content [224]. However, the large number of these enzymes, and the resulting redundancy, means that it is difficult to obtain significant effects simply by altering only one of them. For example, in *Arabidopsis* plants the simultaneous deficiency of three couples of lignin-involved peroxidases (prx2/prx25, prx2/prx71, and prx25/prx71) lowered lignin content much more than in single mutants (without affecting the growth) [225]. Similar multiple mutants (prx2/prx25 and prx2/prx25/prx71) were needed to produce *Arabidopsis* seeds with thinner protective layers and lower polyphenol content and to make them more sensitive to accelerated ageing and more permeable [226].

Similar effects have been recorded with laccases. For example, two laccase double mutants, lac4-1 lac17 and lac4-2 lac17, caused, respectively, a 20% and 40% lignin reduction, while deposition was completely prevented with the triple mutant lac4 lac11 lac17 [147]. 

## 4. Advanced Applications of Lignin-Based Materials

While Section 3 of this article is focused on biosynthesis, here advanced applications of lignin-based materials will be discussed, where mainly technical lignin as source was used. The advantage of utilizing technical lignin is that it is directly available on a larger scale as a by-product of the papermaking and biofuel industries. To utilize lignin for material design, it has to be isolated from biomass using different processes. The different types of technical lignin are briefly discussed here, and we refer the reader to excellent and specialized recent reviews for more in depth covering of this subject [227,228,229]. The method for lignin extraction affects the native structure of lignin through the cleavage of bonds between different lignin monomers and can result in chemical modifications. Here, the content of functional groups in the lignin (phenolic hydroxyl, carboxyl, and sulfonate groups) varies with the pulping process applied. Kraft lignin (KL) is obtained by Kraft pulping. It is the major chemical pulping process, which is performed at high pH value, and the lignin is precipitated from black liquor by the addition of acidifying agents (mineral acid or carbon dioxide) [230]. Depending on the pH value which was utilized, different compositions and yields of the KL can be obtained. Lignosulfonate can be isolated by a sulfite pulping process, which is performed between pH = 2 and pH = 12 (depending on the cationic composition of the pulping liquor). The resulting lignosulfonate includes a high amount of sulfonate groups. Another class of technical lignin is the organosolv lignin. Here, biomass components can be fractionated by means of an organic solvent (ethanol, ethylene glycol, acetone, and tetrahydrofuran). The resulting organosolv lignin is free of sulfur components and provides a higher homogeneity in contrast to KL or lignosulfonate [231]. Soda lignin (or alkali lignin) is obtained by soda pulping or delignification using strong alkali. For this purpose, the biomass is heated in the presence of 13–16% alkali [227]. The main difference in comparison to the Kraft pulping process is the utilization of a sulfur-free medium of the cooking liquor, and for this reason, the soda lignin is sulfur-free [232].

**Figure 7:**
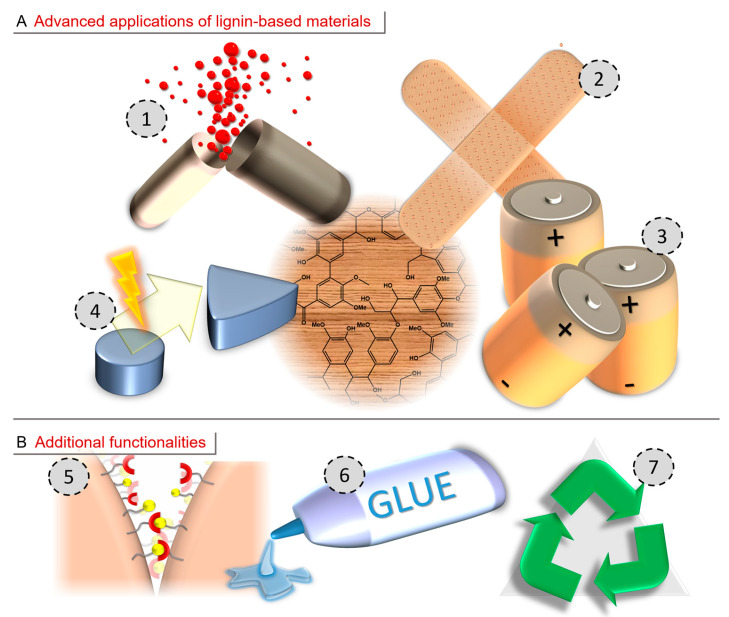
Schematic illustration of (**A**) advanced applications of lignin-based materials and (**B**) additional functionalities, which can be integrated. 1. Drug delivery, 2. wound healing, 3. energy storage, 4. smart materials, 5. self-healing, 6. adhesiveness, and 7. recyclability.

The substantial development in lignin pretreatment and processing technologies has enabled the design of lignin-based materials for advanced applications (Figure 7). In this section, we present how lignin is utilized to highlight its potential for health care applications, for energy storage, and as smart materials, as well as demonstrating how lignin-based materials can be equipped with additional functionalities such as self-healing, recyclability, and adhesiveness. It has to be noticed that lignin-based materials were also successfully integrated into material systems indented for other types of applications, e.g., for the agroindustrial field or as bioplastics, and we refer the reader to specialized recent reviews [233,234,235].

### 4.1. Lignin-Based Materials for Healthcare Applications

Processes of increased sophistication (above all those that allow to obtain sulfur-free lignin) [236] have progressively allowed to obtain lignin with sufficient reproducibility in chemical composition and overall architecture to allow the development of lignin-based materials for applications in the health care sector, including for tissue engineering and drug/gene delivery [237]. It is of note that this is in part due to physical properties and in part to chemical reactivity: the abundance of phenols and catechols enables lignin to act as a reasonably strong antioxidant and therefore scavenge biologically relevant oxidants (ROS, reactive oxygen species), which—although the correct level of scavenging matters—can be instrumental for an accelerated wound healing [238]. In addition, the inherent amphiphilicity of lignin enables the formation of nanomaterials with phenolic (hence a negative charge at physiological pH) and aliphatic alcohols covering the surface and aromatic and methyl groups composing the bulk and thereby allowing for lignin to become the basis for carriers of hydrophobic molecules [239]. Hence, lignin-based particles can be equipped with diverse therapeutics for many pharmaceutical applications including anticancer treatment [240].

#### 4.1.1. Lignin-Based Nanoparticles

On the one hand, lignin and its derivatives can be loaded in nanoparticles (NPs) as presented with particular systems based on dextran and glycol, which were used for in vivo wound healing [241]. On the other hand, lignin-based NPs can be prepared using different synthesis methods, which affect the size distribution [239,242]. Nanoprecipitation is a widely used process to create lignin-based NPs [243]. It is a solvent shifting method, in which supersaturation of a lignin solution (organic solvent is used) is obtained by a controlled shifting into a non-solvent rich medium (e.g., water) resulting in particles with spherical shape, diameter ≥ 100 nm, and negative surface charge. The characteristics of NPs (morphology, size, and surface charge) can be controlled by the parameters of the formation process (solvents, concentration, and type of lignin). 

As example, when alkali lignin (AL) is precipitated under acidic conditions, monodisperse size distribution can be realized [244]. In contrast to that, the precipitation of AL using water results in the formation of heterogeneous particle sizes [245]. A size distribution between 50 and 350 nm could be obtained for NPs, which were generated by the precipitation of lignin (dissolved in organic solvents) in water [246]. Here, NP sizes were affected by the molar mass, content of hydroxy groups, volume of water, and the stirring rate during preparation. The pure lignin-based NPs showed an advanced tissue formation in the treatment of skin wounds as investigated in a mouse model. The particles did not interfere with cell proliferation during wound healing. 

Lignin-based NPs for tissue engineering can be equipped with calcium peroxide (enabling a controlled release of oxygen) for an enhanced vascularization and maturation of collagens [247]. Here, around 500 ppm oxygen per day could be released by calcium peroxide, which was incorporated in sodium lignosulfonate (LS)-based NPs. These lignin composite precursors could be injected together with an alkene-functionalized gelatin matrix, which is photo-crosslinkable. These in situ forming lignin-based composites including NPs (which can release oxygen) improved blood vessel formation and supported the infiltration of smooth muscle actin and fibroblasts into the wound defects. Recently, an accelerated wound healing could be realized with organosolv lignin-based NPs that act as drug nanocarriers [248]. The NPs were loaded with curcumin (wound-healing active, antioxidant, and anti-inflammatory property) as a promising small molecule for the treatment of wounds. In vitro investigations indicated a strong antibacterial activity against Gram-positive bacterial pathogens (*S. aureus*). Fibroblast cell migration was detected using a scratch assay. Here, wounded keratinocytes exhibited increased cell migration upon treatment with curcumin loaded nanocarriers. An enhanced dermal wound closure (nearly full wound contraction after 12 days) in comparison to an untreated control (wound size reduction of 43% after 12 days) could be observed in in vivo experiments using wounded rats.

Drug delivery using pH-sensitive and amphiphilic lignin-based copolymers as carrier materials showed adjustable release profiles as function of pH value [249], using AL functionalized with methacrylate groups. In a second step, graft copolymerization with methyl methacrylate (MMA) was performed resulting in AL-*g*-PMMA copolymers. NPs with a hydrodynamic diameter of about 180 nm could be obtained by self-assembly. When the particles were loaded with ibuprofen as drug, a release of about 82 wt% of the load could be detected at a pH value of 7.4 (simulating intestinal fluid), whereas at a pH of 1.5 (simulating gastric fluid) less than 16 wt% of the drug was released within 72 h. The designed NPs were able to inhibit the survival of human colon cancer cells HT-29 with a final survival rate of only 5.3%.

Lignin-based NPs successfully demonstrated the ability to encapsulate ascorbic acid (AA; can act as antioxidant additive to contrast skin aging processes and to protect the cell against photo-damage) in order to improve stability and antioxidant activity against temperature and pH-dependent degradation [250]. An AA ester was synthesized and was efficiently encapsulated inside NPs from KL by a solvent shifting procedure (KL and AA derivatives were dissolved in dimethyl isosorbide; after addition of water NPs precipitated). The loaded NPs showed improved stability towards temperature and pH changes, and the included AA derivatives were preserved towards degradation. After 24 h, the AA derivatives could be released up to 84% at pH 3.0 and up to 78% at pH 5.4.

The treatment of fungal diseases (exemplarily shown in plants) was investigated with lignin-based nanocarriers with a diameter of 200–300 nm, which were synthesized by miniemulsion polymerization (based on aza-Michael addition reaction) utilizing methacrylated kraft lignin (KL) and bio-based amines as a crosslinker [251]. When fungicides were encapsulated in situ during the miniemulsion polymerization (encapsulation efficiencies between 70 and 99%), the growth of *Phaeomoniella chlamydospora* and *Phaeoacremonium minimum* (lignase-producing fungi) could successfully inhibited (proved for 4 years *in planta* studies).

It has to be noticed that the essential characteristics of lignin-based NPs utilized as drug carriers are the capacity of drug loading and the efficiency of drug encapsulation. As an example, when doxorubicin hydrochloride (DOX) was loaded into lignin-based NPs during NP preparation, better encapsulation efficiency than for introducing the drugs into NP colloidal solution was obtained as result of stronger interactions between lignin and the utilized drug [252]. According to the chemical structure of lignin, strong interactions (hydrogen bonding and π-π stacking) especially with hydrophobic drugs can be realized [253,254,255]. As result of these strong interactions, an extremely slow release profile can be obtained as presented for curcumin loaded lignin-based NPs in simulated gastric conditions (<30% release within 6 days) [254]. Positively charged drugs can interact with lignin-based NPs as they provide a negative surface charge enabling ionic bonding [256], but the drug loading using hydrophilic molecules (such as capecitabine) is still challenging [257]. In addition, the translation of described materials for health care applications requires reproducibility of material characteristics in accordance with structure–property relationships. As the lignin structure (e.g., molar mass, dispersity, and degree of branching) and functionality (e.g., content of aromatic hydroxy groups and aliphatic hydroxy groups) are highly dependent on the lignin source and technical process for lignin production, systematic investigations about chemical composition and macromolecular structure of lignin as function of the different types in reference to drug delivery and tissue engineering characteristics are essential.

#### 4.1.2. Lignin-Based Hydrogels

**Figure 8:**
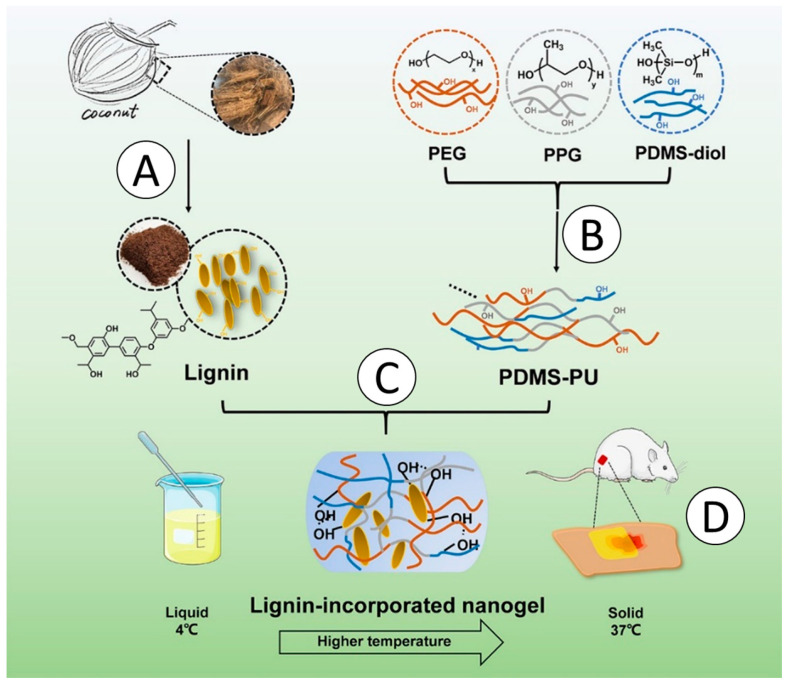
Schematic illustration of the synthesis of lignin-incorporated nanogels for wound healing application. (**A**). Extraction of lignin from coconut husk. (**B**). Synthesis of the thermoresponsive nanogel based on poly(ethylene glycol) (PEG, brown), poly(propylene glycol) (PPG, grey), and poly(dimethysiloxane) (PDMS-diol, blue). (**C**). Preparation of temperature-sensitive lignin-incorporated nanogel. (**D**). Wound healing application of the nanogel. Reprinted with permission from ref. [238] Copyright 2021, American Chemical Society.

The many functional groups of lignin can be employed to integrate lignin fragments in sustainable and environmentally friendly hydrogels, which are recognized for their interesting applicability in biomedicine [258]. As example, an antioxidant hydrogel was created by crosslinking KL (acting as antioxidant agent) and gelatin in water [259]. The hydroxy group from KL and the primary amine groups from gelatin were reacted with epichlorohydrin under alkaline condition, whereby covalent bonds were formed in a ring-opening reaction. These hydrogels showed the reduction of oxidative stress as investigated by in vitro natural antioxidant expression tests and high antibacterial activities against *E. coli*. When AL was crosslinked with agarose to generate hydrogels and extracted silk fibroin (enhancing mechanical tensile properties) as well as when zinc chromite NPs (as anti-infective agent) were incorporated into the hydrogel matrix, lignin-nanobiocomposite scaffolds for wound healing were generated [260]. For these gels, considerable hemocompatibility and antibacterial activity was indicated. An almost completely healed wound within five days was observed in a mouse model. As the desired shape of a biomaterial is always dependent on, e.g., the size and shape of a wound, shape adaptivity can be highly demanded. A lignin-incorporated nanogel was presented, which can be injected as a solution [238]. In that work, a strong antioxidant activity was determined from extracted organosolv lignin (from coconut husks). The lignin was integrated into thermoresponsive nanogels based on polyurethane (PU) copolymers of poly(ethylene glycol) (PEG), poly(propylene glycol) (PPG), and poly(dimethylsiloxane) (PDMS) (Figure 8). These nanogels provided a sol-gel transition between 22 °C and 24 °C (dependent on the lignin content) enabling the formation of a polymeric network at body temperature, which can accommodate the shape of a wound. Here, the burned wound of the mouse treated with lignin-incorporated nanogels showed a complete healing within 25 days (comparable with performance of other antioxidants like vitamin C and trolox) while a slower healing rate was observed when lignin-free nanogels were used.

Lignin-based hydrogels can be utilized as a drug delivery platform. In one example, organosolv hardwood lignin was used to design hydrogels by crosslinking with poly (ethylene glycol) diglycidyl ether (PEGDGE) [261]. As a hydrophilic model drug, paracetamol was selected for release studies. The paracetamol release was affected by the composition of the hydrogels. With increasing crosslinker content, drug release was reduced as result of increasing molecular interactions (hydrogen bonds) between the drug and the crosslinker. However, the composition of the reported hydrogels has to be optimized as they showed a fast burst release profile with paracetamol as hydrophilic drug. The efficiency of lignin-based hydrogels as drug delivery matrix for hydrophobic drugs such as curcumin was investigated with hydrogels synthesized by crosslinking LS lignin with PEG and poly [(methyl vinyl ether)-*co*-(maleic acid)] [262]. The hydrophobic nature of lignin facilitated curcumin loading, and the prepared hydrogels were able to sustain the release for up to 4 days. Another way to incorporate drugs in lignin-based hydrogels was presented by means of generating inclusion complexes when lignin is equipped with cyclic oligosaccharides. Here, crosslinked LignoBoost lignin was functionalized with β-cyclodextrin (β-CD) to create a drug carrier matrix [263]. Active components such as ketoconazole and piroxicam form inclusion complexes in an aqueous environment with β-CD, as β-CD provides a hydrophobic core cavity. The obtained drug carriers showed fast drug release kinetics (>80% release within 10 h), which fitted well in the Korsmeyer–Peppas model. Although great progress was made for lignin-based hydrogels intended for drug delivery, the ability of changing the swelling behavior when external stimuli are applied to obtain an on-demand release would be important to facilitate the transition from fundamental research into real health care applications.

#### 4.1.3. Lignin-Based Foams

An additional application area for lignin is the design of foam materials, as they provide a low density, which leads to lower material costs. Lignin-based polyurethane foams (PUFs) were reported, which effectively promote wound healing of full-thickness skin defects [264]. In a first step, enzymolysis lignin was functionalized with PEG and glycerol. Subsequently, a one-step foaming process was applied with a silver nitrate solution and hexamethylene diisocyanate. Silver nanoparticles were created in situ during the foaming process as the phenolic hydroxy groups included in the liquefied lignin acted as reducing and capping agent to silver ions. The created PUFs exhibited more than 99% antibacterial rate against *E. coli* within 1 h and *S. aureus* within 4 h. An increase of the silver nitrate concentration for the forming procedure increased the antibacterial ability of PUFs, indicating that the silver nanoparticles are significantly contributing to the effect against bacteria. Evaluations in a mouse wound healing model indicated that the designed lignin-based foams could effectively promote wound healing of a full-thickness skin defect.

Lignin-based materials with a 3D structure were obtained by means of coaxial electrospinning resulting in scaffolds for wound dressing application [265]. Scaffolds formed from core–shell fibers based on a chitin–lignin gel (as core using Biolignin^TM^) with PCL (as shell). The presence of a PCL shell layer decreased the dissolution time of the chitin–lignin gel fiber and provided in this way sustainable release of methylene blue, which was utilized as model drug. When penicillin and streptomycin (showing bacterial inhibitory effect) were introduced in the lignin-based gel, a clear inhibition zone against *S. aureus* and *E. coli* bacterial strains could be obtained.

As the properties of lignin-based foams/scaffolds are highly related to the 3D structure (pore size, density, and wall thicknesses), the processing can determine the characteristics of the materials [266]. Hence, investigations focusing on the impact of micro-/nano-porous structure of lignin-based foams on wound healing ability and drug delivery capacity would be of interest.

#### 4.1.4. Multifunctional Lignin-Based Materials

An additional feature of lignin-based materials for health care applications was described for lignin/poly(ionic liquids) composite hydrogels utilized as dressing, which provided self-healing properties [267]. The mechanical strength of the hydrogel dressing was effectively improved by the introduction of lignin while the poly(ionic liquid) based on (3-butyl-1-isopropyl-1H-imidazol-3-ium bromide) enabled good antibacterial activity and self-healing. As result of supramolecular interactions within the lignin/poly(ionic liquids) compounds, the dressing could be reused many times also after simple high-temperature disinfection.

Lignin-based materials showing accelerated wound healing and providing repeatable adhesiveness to a variety of substrates were described using Ag-lignin (AL was used) NPs loaded in a poly(vinyl alcohol) (PVA) hydrogel matrix together with cellulose nanocrystals [268]. Here, phenol or methoxy groups in lignin can reduce silver ions to metallic silver NPs, while functional lignin groups will be oxidized to the corresponding quinone or hydroquinone. The conversion of these groups into catechol groups is enabled in the presence of photogenerated electrons (provided by Ag NPs) [269]. The hydrophilic hydroxy groups of PVA could interact with various surfaces, whereas the generated catechol groups provide strong adhesion and quinone groups can create physical cross-links with PVA and enhance cohesion.

**Figure 9:**
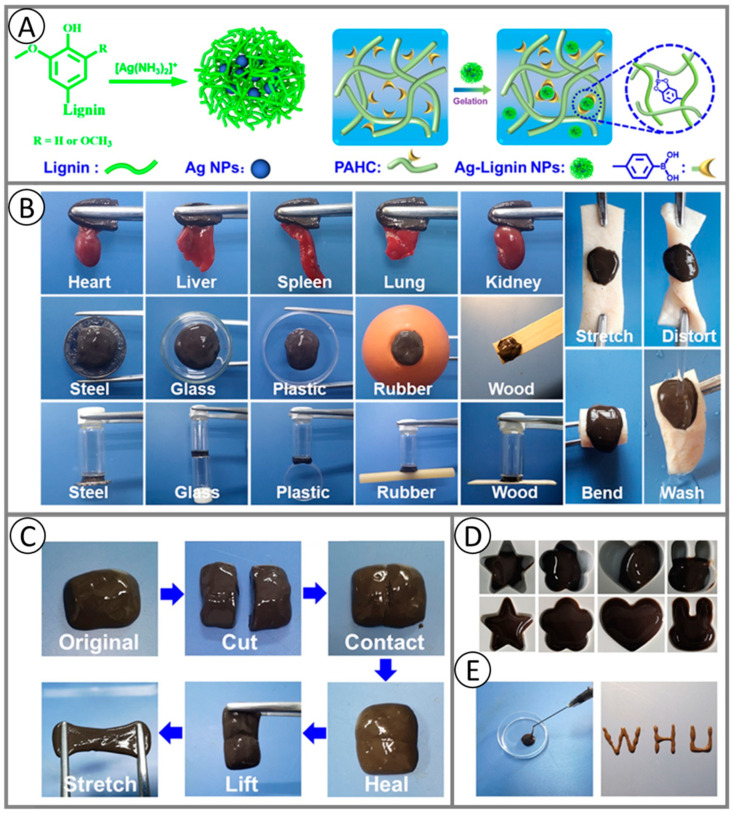
(**A**). Synthesis of multifunctional hydrogels as wound dressing materials based on lignin (light green), Ag nanoparticles (blue), and phenylboronic acid-modified hydroxypropyl cellulose (PAHC, gren). (**B**). Adhesion on organic and inorganic substrates. (**C**). Self-healing capability, (**D**). shape adaptivity, and (**E**). Demonstration of injectability of the created lignin-based hydrogels. Adapted with permission from ref. [270]. Copyright 2021, American Chemical Society.

A strong adhesion capability was determined for multifunctional hydrogels created by crosslinking of phenylboronic acid-modified hydroxypropyl cellulose (PAHC) using Ag-AL NPs (lignin reduced in situ a [Ag(NH_3_)_2_]^+^ complex resulting in Ag-lignin NPs) (Figure 9) [270]. The introduced particles acted as antibacterial nanostructure and crosslinking agent (catechol of lignin can form dynamic borate ester bonds with PAHC). The phenolic hydroxy groups endowed lignin with a strong adhesion capability, and the complexation of lignin and Ag can generate a quinone/catechol structure (dynamic redox environment) enabling a repeatable adhesion. Therefore, the designed materials could adhere to a variety of organic (including wound tissue) and inorganic substrates, could close a wound surface, and could achieve a good hemostasis effect. As the hydrogel dressings included dynamic borate ester bonds and hydrogen bonding, an excellent self-healing ability could be demonstrated. The materials provided a shear thinning, which enabled hydrogel injection as well as the adaption of the hydrogel system to a mold shape.

A high potential for the application in personalized healthcare was addressed by multifunctional on-chip electrochemical sensors, which were obtained by a laser-scribing process that converts lignin-based (lignosulfonate) precursors into conductive nitrogen-doped graphene patterns [271]. These electrodes were modified with an MXene/Prussian blue composite and with catalytic enzymes for selective detection of glucose, lactate, and alcohol (markers of diabetes and indicator of athletic performance and of alcoholism) resulting in an enhanced electrochemical activity toward the detection of these biomarkers.

As lignin and lignin-based NPs are of interest for the health care sector and in addition could act as a platform for the immobilization of different enzymes as presented with the example of peroxidases [272], we believe that new types of multi-functional lignin-based materials will be designed considering also enzyme supported pathways to treat skin diseases or infections in near future.

The strategies of how different functions (self-healing, adhesiveness, shape adaptation, and biochemical sensing) can be introduced in lignin-based materials open up a new direction of high value-added products for health care applications and will hopefully inspire other scientist to utilize lignin as source for multifunctional biomaterials.

### 4.2. Supercapacitors Based on Lignin

Supercapacitors are one of the most effective and practical technologies for energy storage. They enable the spanning of the power/energy gap between batteries and conventional dielectric capacitors by providing a short charge time, high power density, and long cycle life [273]. The underlying energy storage mechanisms are connected to reversible faradic reactions or to electrostatic charge accumulation (at the interface of electrolyte/electrode). Porous carbon materials, carbon based nanotubes, and ordered mesoporous carbon materials were considered to be the most suitable electrode materials for supercapacitors due to their high specific surface area, developed pore structure, high electronic conductivity, and excellent stability [274]. In comparison to other common precursors such as polyaniline, pitch, and rayon, the most suitable precursor for the design of high-performance carbon fibers is polyacrylonitrile (PAN), as it provides a high melting temperature (*T*_m_), provides high carbon content, and can be pyrolyzed very quickly resulting in fine and regulated fiber diameters. As disadvantage, PAN and other petroleum-derived polymers are expensive and release toxic components during a carbonization process [275]. Hence, lignin as green carbon source for the development of supercapacitors presents a promising alternative [276].

#### 4.2.1. Lignin as Electrode Material

Lignin fragments are not easily spinnable into fibers as they have a low molar mass. For this reason, polymers with a high molar mass (PAN, PEG, and PVA) can be added to a lignin solution, whereby viscosity and spinnability can be improved [274]. Organosolv lignin can be used for supercapacitor applications as it includes a low ash content resulting in carbons with very few metal impurities (which have to be removed for long-term cyclability). A lignin-derived carbon nanofiber (CNF) electrode was created by electrospinning of a lignin/PEG solution (90 wt% organosoly hardwood lignin) [277]. The obtained CNF mats were densified by uniaxial compression (between 40 and 120 bar) and were carbonized at 800 °C. The densification occurred by reducing the inter-fiber pore size. The improvement of packing density was directly proportional to the performances of the created carbon nanofiber electrode (reaching a volumetric capacitance of 130 F·cm^−3^ and energy density of 6 Wh·L^−1^ at 0.1 A·g^−1^).

Conductivity of lignin-based electrode materials can be improved upon by integration of polypyrrole particles into the electrode material. Lignin/polypyrrole composite electrode films were created with microporous and mesoporous structures by means of electrospinning, carbonization, and in situ polymerization methods [274]. The specific surface area could be increased (up to 872.60 m^2^ g^−1^) by carbonization, which induced the removal of carbonyl and phenolic functional groups of lignin. Afterwards, polypyrrole particles were added to the lignin nanofibers. The synthesized lignin/polypyrrole composite anode provided good electrochemical performance with a large specific capacitance of 213.7 F·g^−1^ (at a current density of 1 A·g^−1^) in 1 M H_2_SO_4_ as the electrolyte. The incorporation of multi-channels in lignin-based CNFs enabled the design of high-performance energy storage devices with an excellent cycling stability (5% capacitance decay over 10,000 cycles at 10 A·g^−1^) [278]. Here, CNFs nanocomposites were synthesized by means of co-electrospinning using poly(pyrrolidone)-SnCl_2_·2 H_2_O as a shell material and lignin/PMMA as core materials. A following heat and acid treatment resulted in a pore-forming effect of SnCl_2_·2 H_2_O and the generation of SnO_2_. When the lignin/PMMA composition was optimized, nanocomposites with hierarchical internal channels, porous surface, and high specific surface area could be produced. Besides the great cycling stability, supercapacitor electrodes based on these type of CNFs nanocomposites exhibited high specific capacitance (406 F·g^−1^ at current density of 0.5 A·g^−1^).

Instead of electrospinning, a dual template technique can be used for the design of porous lignin-based materials with supercapacitor application [279]. A hierarchical porous carbon monolith was fabricated via dual templates (Pluronic P123 and silica NPs) with a following carbonization procedure. When silica NPs of different sizes and amounts were utilized, the resulting 3D structure of the carbon monolith could be tuned. For an optimized porous morphology (using NPs of 7 nm) good electrochemical performance was realized. The symmetric cell assembling with this type of carbon electrode resulted in a large amount of energy (131 μWh·cm^−2^) at high power densities (1368 μW·cm^−2^) and good cycle stability (~93% after 10,000 cycles). Porous lignin-derived carbon quasi-nanosheets intended for the application as a supercapacitor were designed by a green and facile in situ carbonization technique [280]. For this purpose, industrial waste LS/zinc oxalate composites were fabricated from ethanol/water solution, and carbon quasi-nanosheets could be obtained by a following co-pyrolysis with gas-exfoliation and templating of zinc oxalate. For such materials, a long cycling stability (93.5% of the original capacitance after 10,000 cycles at 5.0 A·g^−1^) could be realized. An excellent electrochemical performance (high specific capacitance, excellent rate capability, and high specific energy density) was shown when the prepared materials were assembled into symmetric supercapacitors in PVA/KOH gel electrolytes.

As promising method for the design of lignin-derived electrodes, direct laser writing was introduced [273]. Laser induced graphene was synthesized from KL via direct laser writing resulting in the formation of a hierarchical structure with a 3D interconnected network. Laser writing in this process photothermally converts KL into few-layered graphene. Soft electrodes could be fabricated by transferring the created few-layered graphene onto PDMS. The produced flexible supercapacitors exhibited good electrochemical performance and good cyclic stability (>90% capacitance was retained after 10,000 cycles). As an advantage of their flexibility, the supercapacitors were able to withstand bending deformations without significantly losing capacitance.

An important upgrading step of lignin-based materials for energy storage application is the physical or chemical activation to enlarge surface area and facilitate efficient permeation of the electrolyte [281]. Physical activation can be performed with carbon dioxide, steam, or a combination of them. The creation of pore structures includes a pore drilling mechanism, which increases the diameter of pores, and a pore deepening mechanism, which influences the pore depth [282]. While carbon dioxide activation will result in the formation of micropores, meso- and macropores can be obtained by means of steam activation [283]. An improved quality consistency and shorter residence time required for activation can be realized by chemical activation through incorporation of, e.g., KOH, H_3_PO_4_, or ZnCl_2_ in lignin-based materials [281]. As an example, a regular and well-developed porous network was formed by the incorporation of ZnCl_2_, as it can diffuse throughout the carbon matrix (molten above 290 °C) and can migrate through the microporous network at high temperatures (boiling point ~730 °C) [284]. The effect of different chemical activation components, which were impregnated on AL, on porous structure and resulting electrochemical properties was analyzed [285]. Here, ZnCl_2_- and KOH-activated carbons contained mesoporous structures, while K_2_CO_3_ as activating agent resulted in the formation of micropores. For the series of activated-carbon materials, a specific capacitance of 142.09, 251.04, and 263.46 F g^−1^ for an activation using ZnCl_2_, KOH, and K_2_CO_3_, respectively, was obtained.

A carbonization step or thermal treatment is not necessary for the design of lignin-based electrode materials as reported for CNFs with an inter-fiber bonding structure. Here, CNFs were developed by esterification of organosolv lignin with butyric anhydride followed by electrospinning with PAN [286]. The resulting nitrogen–oxygen co-doped CNFs provided a high heteroatom content and a good wettability. Lignin-based CNFs were utilized as electrode materials, which showed a high specific capacitance (320 F·g^−1^ at 1 A·g^−1^ with 6 M aq. KOH as electrolyte). A high coulombic efficiency of 112.5%, good energy density of 17.92 Wh·kg^−1^, and good cycling stability (<6% loss after 5000 cycles) were obtained when assembled CNFs//CNF symmetric supercapacitors were analyzed. 

It has to be noticed that the specific surface area and pore size distribution is determining the performance of porous carbon materials for energy storage. As lignin provides a complex molecular structure, the pyrolysis behavior and the design of electrode materials are only limitedly controllable. In addition, the molar masses and content of impurities are dependent on the lignin source and type of production, which will influence the physicochemical properties of the final product [287]. Accordingly, a uniform strategy for the development of lignin-based supercapacitors could not be achieved the inconsistency of industrial lignin needs to be further considered; and the relationship between lignin structure/resource and product properties have to be systematically investigated.

#### 4.2.2. Lignin as an Electrolyte Material

As specific functional groups within the lignin structure (benzyl and phenolic groups) could act as active reaction sites for ions, lignin-based materials are of interest for the design of electrolytes. In addition, the numerous present oxygen atoms are important for promoting electrolyte ion adsorption and redox reactions [230]. Hence, an approach to utilize lignin for potential application in energy storage devices is the creation of lignin-based hydrogels as electrolytes, which are flexible and compression-resistant [288]. A double-crosslinked lignin hydrogel synthesized by crosslinking lignin and forming hydrophobic lignin aggregates (obtained when the hydrogel is treated with H_2_SO_4_ solution) was used to create lignin-based hydrogels serving as electrolyte. In combination with polyaniline-deposited carbon cloth as the electrode, a high specific capacitance of 190 F·g^−1^, excellent energy density, and good cycling stability were realized. This flexible supercapacitor was able to retain high specific capacitance after 500 cycles of 180° bending or 80% compression strain. As lignin can be used as electrolyte material and as electrode material, the supercapacitors based on a lignin-derived hydrogel electrolyte and a lignin-based electrode were investigated [289]. The chemically cross-linked AL hydrogel electrolytes were synthesized by a base-catalyzed ring-opening polymerization and crosslinking reaction with poly(ethylene glycol) diglycidyl ether. The obtained hydrogels were flexible, provided dimensional stability even in a highly swollen state (523% swelling capacity), and had high ionic conductivity (10.35 mS·cm^−1^). Electrodes were fabricated by electrospinning using a mixture of lignin with PAN in DMF followed by a stabilization process in air at 250 °C (formation of an N-containing ladder-type structure). The resulting stabilized nanofiber mats were carbonized at 900 °C and provided interconnected porous channels. The created supercapacitor devices (including lignin hydrogel electrolyte and electrospun lignin/PAN nanofiber electrodes) demonstrated a high capacitance (129.23 F·g^−1^) and capacitance retention (95% over 10,000 cycles), demonstrated flexibility and durability under diverse bending angles, and delivered a maximum energy and power density of 4.49 W·h·kg^−1^ and 2.63 kW·kg^−1^, respectively. The mentioned examples of how lignin can be utilized for energy storage application highlight promising strategies to reduce product costs and to contribute to the development of more sustainable and greener energy devices. Unfortunately, the practical application of such lignin-based materials is still greatly limited, but the research progress is an important step toward green energy technology.

### 4.3. Lignin-Based Shape-Memory Materials

**Figure 10:**
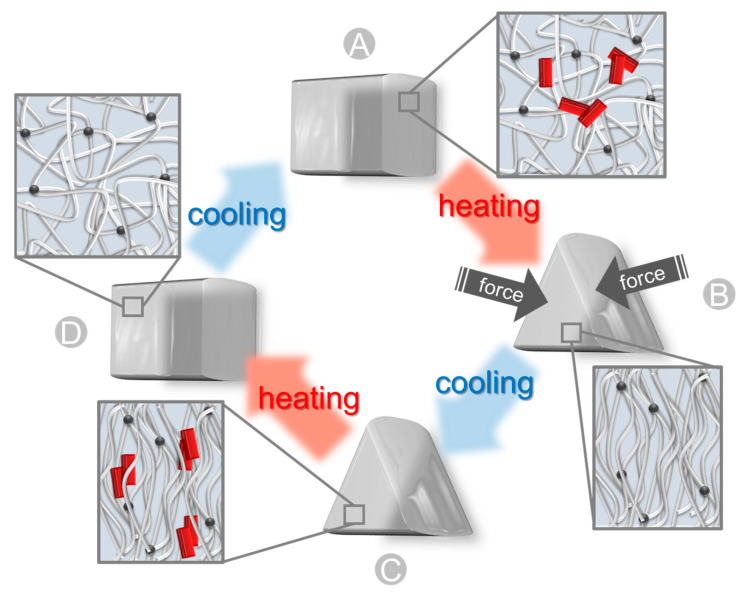
Shape-memory effect; here shown for a material using crystallization for fixing the temporary shape, though other options exist. The original shape (**A**) of a material is heated above the melting temperature of crystalline domains (red, acting as temporary netpoints). Then, the material can be deformed into shape (**B**) resulting in the orientation of the polymer chains. When the temperature is decreased, crystallization is induced, and the temporary shape (**C**) can be obtained after removal of external stress. The recovery process is initiated by temperature increase enabling the polymer chains to regain their random coil formation. As result, a directed movement to the original shape (**D**) can be realized.

The next generation of value-added applications is the development of lignin-based smart materials. Such materials can convert specific external stimuli to defined outputs and change or activate a functional property of the material [290]. Properties can be designed to change in a controlled fashion when, for example, temperature, moisture, pH, or electricity are applied [291]. Shape-memory polymers (SMPs) especially have received an increasing attention because of their potential applications in biomedical treatment, sensors, and actuators [292]. These kinds of materials are capable of translating changes in the environment into a directed geometric movement. The resulting function of the material is denominated as a shape-memory effect (SME). To activate an SME, a stimulus as an input signal causes the removal of an internal structural barrier (created during a programming procedure) and initiates the recovery of the original shape [293,294]. As an example, the programming process of a material providing shape memory properties involves a shape deformation (e.g., in an amorphous state of the material), a shape fixation (obtained when temporary crosslinks are generated, e.g., when crystallization is induced by decreasing the temperature), and the release of the external stress (Figure 10). As result, a higher entropic state is created as polymer chains which will be oriented according to the deformation direction (stretched polymer chains), and the movement of polymer chains back to their random coil formation is blocked by the generated temporary crosslinks. Finally, a directed movement back to the original shape (driven by entropy elasticity) can be realized when a stimulus is applied (e.g., heat).

#### 4.3.1. Temperature-Induced SME

Lignin can be utilized as crosslinker in SMPs. Thermosets based on KL (as a crosslinker), PEG (*M*_n_ = 400 g·mol^−1^, creating temporary netpoints), and citric acid (acting as a crosslinker) were synthesized by a green one-pot heat curing method, in which only water was produced as byproduct [295]. An increase of the lignin content (from 20 to 40 wt%) resulted in increasing crosslinking density, which drastically increased the storage modulus from 5.7 MPa to 2 GPa and the glass transition temperature (*T*_g_) from −0.3 °C to 102 °C. Excellent shape-memory properties with a shape fixation of 95% and a thermally induced shape recovery of 99% at 80 °C (when 35 wt% lignin was utilized) could be obtained. Instead of PEG, acrylonitrile butadiene rubber can be crosslinked with organosolv hardwood lignin [296]. The thermal annealing at 180 °C results in the creation of reactive sites within lignin promoting crosslinking reactions, and the *T*_g_ of the rubber matrix could be enhanced by 18 °C. Shape-memory experiments were carried out with a deformation of the lignin-based composites at 100 °C, a fixation at 0 °C, and the thermally induced recovery at 50 °C and/or 100 °C. In this case, tunable chemical and physical crosslinks within lignin and the utilized rubber resulted in good shape programmability and a quick shape recovery. Directed shape shifts utilizing a physiological relevant temperature range could be realized by means of PUs based on castor oil, LS, and sophorone diisocyanate [297]. Here, the implementation of LS into the PUs increased the *T*_g_ from 9.7 °C (pure castor oil) to 18.6 °C (3 wt% LS). The designed PU-LS films could be programmed at 37 °C and showed an excellent SME with a recovery to the original state within 17 s at 37 °C. 

In contrast to utilizing lignin only as crosslinker, it can be incorporated in polymeric networks to provide temporary netpoints as demonstrated for lignin-based PUs with 100% biobased carbon content [298]. Here, non-isocyanate PUs were created using a non-toxic cyclocarbonation scheme with KL. First, unmodified KL was oxyalkylated using glycerol carbonate. In a subsequent step, the resulting 1,2-diol functionalized macromolecule underwent a transesterification reaction with dimethyl carbonate resulting in the formation of cyclocarbonate groups. Finally, the PUs were obtained after the addition of a fatty acid-based dimer diamine as curing agent. The resulting materials possessed a broad temperature transition region (related to lignin and to the dimer diamine) between the glassy and rubbery state (0–100 °C) indicating numerous microstates of the polymeric material, which could be related to the large range of molar masses within the utilized lignin (PDI = 4.1). When the PUs were bent at 105 °C and cooled to RT, the deformation was maintained. The reheating under stress-free conditions initiated the shape-memory recovery process to the original shape.

As different lignin sources could provide a high difference in functional groups, material properties will be affected. In this context, it was detected that the miscibility of lignin with a polyol for PU synthesis correlated with the number of functional groups (COOH and aliphatic OH) within different types of lignin (softwood KL, organosolv pine bark lignin, and organosolv oak bark lignin) [299]. It has to be noted that the efficiency of the crosslinking reaction for PU synthesis depends on the ratio and reactivity between isocyanate groups and alcohol groups. As result of a lower reactivity of aromatic OH groups with isocyanates, a less uniform cellular structure in PUs was reported resulting in lower compressive moduli when lignin with a high content of aromatic OH groups was used. While softwood KL with a content of aromatic OH groups of 3.75 mmol·g^−1^ showed a compressive modulus <0.4 MPa, organosolv oak bark lignin had a lower aromatic OH content of 3.12 mmol·g^−1^ and provided a compressive modulus >0.6 MPa. The resulting PUs exhibited an increase of *T*_g_s by about 20 °C (dependent on the lignin source, *T*_g_s between 120 and 160 °C were obtained), excellent shape fixation (100%), and good shape recovery behavior (≥80%). As material properties are also highly dependent on molar mass and branching degree, the investigation of the structure–property relationship for lignin is complicated. In this context, a promising strategy using a solvent fractionation of technical organosolv hardwood lignin was followed to separate lignin molar masses and functional groups based on different solubilities [300]. Acetone-soluble fractionated lignin provided a high amount of aliphatic moieties and an aromatic structure with a low branching degree. In contrast, a higher molar mass was determined for the insoluble lignin fraction. In this way, lignin functionalities and physical characteristics can be enriched enabling the control of thermomechanical properties and shape-memory performance in lignin-based multiphase polymers. The fractionated lignins were reacted with acrylonitrile butadiene rubber in the melt-phase to generate partially crosslinked elastomers. Elastomers including the soluble lignin fraction showed enhanced thermal stability (and an increase of *T*_g_ by about 19 K when lignin is added), which was related to strong molecular interactions between lignin and the rubber. Hence, a less pronounced influence on thermal properties (increase of *T*_g_ by about 4 K) was detected when the insoluble lignin fraction was incorporated in the rubber matrix. By means of high-resolution SEM measurements, large phase separation and poor interfacial molecular interactions between the insoluble lignin fraction and the rubber were detected. In contrast, elastomers including the soluble lignin fraction showed a good dispersion of small phase-separated lignin domains within the matrix. The shape-memory performance was affected by the type of integrated lignin. Elastomers with the soluble lignin fraction demonstrated an excellent shape fixation at ambient temperature (after programming at 70 °C, 5% strain loss was measured), whereas a strain loss above 20% was detected for the material including the insoluble lignin component. An almost complete recovery process of both systems was obtained when the temperature was increased to 150 °C. 

#### 4.3.2. Light-Induced SME

The molecular structure of lignin contains a large number of aromatic rings and conjugated functional groups. Hence, strong conjugation and π–π molecular interactions among lignin molecules can be created enabling lignin-based materials with unique optical properties including aggregation-induced emission, UV absorbance, and great potential for sustainable photothermal conversion [301]. Copolymerization of enzymatically hydrolyzed lignin (EL) with epoxy soybean oil (ESO) and PEG gave materials with increased tensile strength from 11.3 to 30.8 MPa and *T*_g_ (93 °C to 115.7 °C) when increasing the EL content [302]. In dependence of the EL content, simulated solar irradiation resulted in an increased surface temperature of the copolymer network as lignin has an excellent photothermal property (can convert light energy into heat). As result of this indirectly induced heating process, directed movements of the materials could be obtained within 20 s (for an EL content of 50 wt%) with excellent shape-memory properties (fixation and recovery >97%). Furthermore, lignin-based castor oil-derived polyamide elastomers were reported, which provided a stretched-induced crystallization of the polyamide elastomer (enables the fixation of a macroscopic shape) and a shape shift triggered by indirectly induced heating processes when near-infrared (NIR)-light was applied [303]. A surface temperature of 200 °C could be obtained after 5 s of NIR laser irradiation. In addition, when standard sun irradiation (100 mW·cm^−2^) was utilized, a thermoelectric generator could be powered. As an application for a light-induced SME for lignin-based materials, an information encryption device was reported [304], Here, lignin was embedded in a cellulose acetate (providing stimuli-sensitivity) matrix. Lignin was utilized as a photo-thermal converter and enabled a rapid photo-thermal response. An information carrier could be realized by a two-step programming procedure at 170 mW/cm^2^ and 90 mW/cm^2^, in which two different temporary shapes could be stabilized by cooling and alternating the light source. The encryption was realized stepwise (two recovery processes-triple-shape effect) by a photo-assisted shape-memory behavior.

#### 4.3.3. Water-Induced SME

Water uptake may trigger shape recovery in SME by cleaving the temporary netpoints, e.g., by acting as softener reducing the *T*_g_. Lignin can be linked to water-responsive shape memory materials in order to avoid the loss of wet strength during excessive hydration [305]. For this purpose, a cellulose nanofiber nanocomposite membrane was prepared including covalently crosslinked PVA and dealkali lignin. The lignin and PVA addition enabled the regulation of water responsiveness and wet mechanical properties (dependent on the lignin content, tensile modulus between 60 MPa and 0.7 GPa could be reached in the swollen state). A recovery of 100% could be obtained within 4 s for a water-induced SME. In addition, the nanofiber-based nanocomposites including lignin provided excellent UV blocking performance with a transmittance value of the membrane <8%. Water responsiveness can further be realized using sodium LS as swelling segment. Here, a 3D porous network with an aligned wall structure was formed in a hydrothermal treatment of single-wall carbon nanotubes and sodium LS (creating a lignin-based hydrogel) [306]. During a vacuum-assisted filtration process, a dispersion of LS and holey graphene oxide gradually penetrated the hydrogel. After a second hydrothermal treatment (resulting in the generation of reduced graphene oxide) and freeze drying, lignin-based ultralight aerogels with a density of 6.9 mg·cm^−3^ were obtained. The aerogels exhibited an excellent shape-memory performance. When the lignin-based aerogel was compressed to 86.2%, a temporary shape could be obtained as result of a high densification degree. Once, the aerogel was placed in water at 25 °C, 94% of its initial height could be recovered within 8 s, and a hydrogel was generated. Here, the hydrophilic LS enabled the swelling, and when enough water filled the pores of the aerogel, the shrunken walls stretched back again into the aligned walls. The aerogel was utilized as water-driven artificial muscle. The demonstrator showed a powerful driving force and could lift 1030.6 times its own weight, which was attributed to the strong aligned wall structure. When the prepared aerogel was utilized as a pressure sensor, a high sensitivity of about 2.28 kPa^−1^ and a wide detection region of 0.27–14.1 kPa could be detected. In addition, the aerogel was assembled into a symmetric flexible supercapacitor with a cellulose/H_2_SO_4_ hydrogel electrolyte. Here, excellent stored energy performance that can tolerate 5000 cycles of bending was shown. 

#### 4.3.4. Multifunctionality in Lignin-Based Shape-Memory Materials

**Figure 11:**
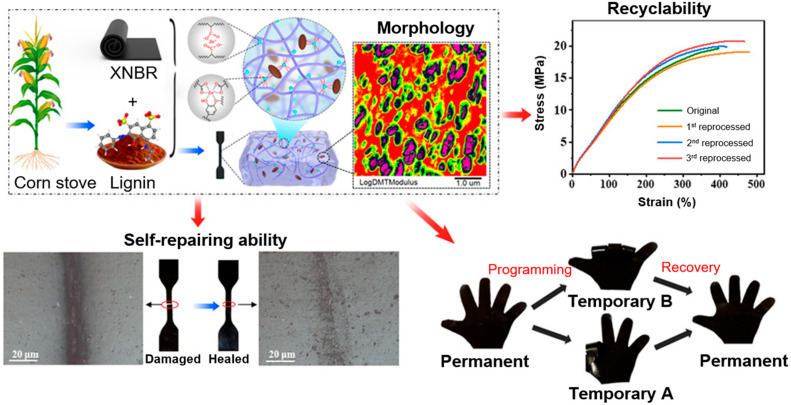
Multifunctionality in lignin-based thermoplastic elastomer composites. Reprinted with permission from ref. [308] Copyright 2022, American Chemical Society.

An additional feature of lignin-based shape-memory materials was reported for a new kind of PUs based on a polyhydroxyurethane including EL, which were prepared using a green and environmentally friendly method (isocyanate-, solvent-, and catalyst-free) [307]. The designed material system possessed a dual network structure consisting of a dynamic covalent network and a hydrogen bonding network. In this case, the lignin-based PUs provided multifunctional characteristics including reprocessability/recyclability, self-healing capability (crack width of a scratched film can heal 90% in 10 s), and thermally induced shape-memory function. The ability of directed movements was attributed to the dynamic covalent network in PUs and based on thermally triggered trans-carbamoylation exchange reactions (carried out at 160 °C). Potential applications as smart packaging label fabrication (for heat-sensitive commodities) and as paper-based electromagnetic shielding materials (when combined with natural cellulose paper) were demonstrated. Metal–ligand coordination bonds present an alternative to induce multifunctional characteristics in lignin-based materials (Figure 11) [308]. An ionomeric elastomer composite including commercial carboxyl elastomer and a high content of EL provided recyclability, self-repair, and an SME. The addition of zinc oxide enabled the creation of metal–ligand coordination bonds between the elastomer (carboxyl group) and the EL (oxygen-bearing groups). As result of the reinforcing effect of lignin and the generated coordination bonds, excellent mechanical properties could be obtained (tensile strength up to 19.6 MPa and elongation at break up to 397%). As the addition of zinc oxide enabled the creation of ionic bonds and ionic aggregates acting as physical crosslinks, recyclability was realized (mechanical properties could be retained or were improved after reprocessing cycles). The ionomeric elastomer composites provided good self-repairable ability. After repairing at 100 °C for 2 h, samples could recover ≥90% of the initial tensile strength and ≥85% of the initial elongation at break. A thermally induced SME could be obtained as a third functionality. While the ionic crosslinks (interaction between Zn ions with the elastomer) acted as permanent netpoints, coordination interaction (elastomer–Zn–lignin bonds) acted as temperature-sensitive temporary crosslinks. Here, the directed movement to the original shape could be enabled within 80 s using a temperature of 80 °C with a fixation and recovery ratio above 85%.

Multifunctional lignin-based materials providing light sensitivity were realized based on maleic anhydride grafted polyethylene elastomer and AL as an efficient photo-thermal agent [301]. Electron transitions from low-energy orbitals to high-energy states were possible by the conjugated structures in lignin resulting in a high photothermal conversion efficiency of 53.7% (a temperature of about 280 °C was reached under NIR irradiation). The smart elastomer composites were able to provide excellent light-controlled self-repairing properties (self-repairing efficiency regarding the tensile strength reached 98.2% within 20 min), a light-triggered shape-memory performance with good fixity and recovery ratios above 80%, and strong photothermal bactericidal activity. Hence, possible applications as smart materials for precise remote driving of robots, machines, sensors, sterilization, and self-repairing equipment were suggested.

In summary, lignin has emerged as a promising option for the design of shape-memory materials with advanced applications as result of its diversity in functional properties. Lignin-based materials are not limited to a temperature trigger to initiate directed movements; also, water- or light-induced shape shifts could be realized. Hence, SMPs including lignin as natural resource are comparable in terms of versatility to conventional smart materials. Although often the stimuli-sensitivity is not directly provided by lignin, lignin significantly contributes to the shape shifting output (e.g., by adjusting *T*_g_ to a physiologically relevant range or by converting light into heat). Thus, research on lignin-based materials with a SME and with SME-related applications will continue rapidly. Not all published work contains sufficient information about the chemical structure of the lignin used. This is a major drawback when trying to establish structure–property or structure–function relationships. It is highly recommended to authors and reviewers in this field to ensure that such information is always included in published work. 

## 5. Conclusions

Lignin structure is, due to the variable biosynthesis, alternative pathways, and partially stochastic reaction mechanism, complex. This complexity is at least retained when extracting it, and extraction processes may add further facets as different parts of wood and different types of woods, which may significantly differ in lignin composition, are mixed. The detailed first part of this article summarizes lignin occurrence and biosynthesis pathways, including the synthesis and structures of canonical and non-canonical monolignols and lignin–polysaccharide hybrid structures. Lignin-based materials have been used in advanced applications exploiting lignin’s complex molecular structure with a variety of functional groups that show antioxidant and antimicrobial properties. We summarized the latest approaches for its use for health care applications, in energy storage devices, and for the design of smart materials such as SMPs. Especially for wound healing and drug delivery applications, much of the performed research is still at the proof-of-concept stage, and the medical products have to be approved by regulatory bodies. As the lignin structure (e.g., molar mass, dispersity, and degree of branching) and its functionality (e.g., content of aromatic hydroxy groups and aliphatic hydroxy groups) are highly dependent on the lignin source and technical process for lignin production, systematic investigations about chemical composition/macromolecular structure of lignin as function of the different types in reference to drug delivery and tissue engineering characteristics are essential before pursuing clinical trials. We believe that the implementation of lignin as a natural source for more technical related applications such as supercapacitors presents a promising strategy, which can be translated into real products in the near future. As the electrochemical performance of these kind of bio-based energy storage devices depends on pore characteristics, surface chemistry, and the structural features of the utilized lignin, a uniform strategy for the development of lignin-based supercapacitors should be considered, and the relationship between lignin structure/resource and product properties has to be elucidated. It is also of importance to attract industrial activity for these new materials, to demonstrate the scale-up, and to better predict batch to batch properties to realize standardized lignin-based products. Last but not least, there is lots of potential for future work to incorporate multiple stimuli in smart materials to address switching “on” and “off” a corresponding lignin-based device. Here, one focus could be how stimuli-sensitivity can be directly provided by lignin to reduce integration of non-bio-based components in order to increase the green fingerprint.

## Data Availability

Data sharing not applicable.
